# Androgen receptor knockdown enhances prostate cancer chemosensitivity by down‐regulating FEN1 through the ERK/ELK1 signalling pathway

**DOI:** 10.1002/cam4.6188

**Published:** 2023-06-16

**Authors:** Weijie Xie, Shulin Li, Huan Guo, Jiawei Zhang, Menjiang Tu, Rui Wang, Bingling Lin, Yuqi Wu, Xiangwei Wang

**Affiliations:** ^1^ Department of Urology and Carson International Cancer Center, Shenzhen University General Hospital and Shenzhen University Clinical Medical Academy Center Shenzhen University Shenzhen People's Republic of China; ^2^ Department of Urology Affiliated Hospital of Guangdong Medical University Guangdong Province Zhanjiang People's Republic of China; ^3^ Department of Radiology Peking University Shenzhen Hospital Shenzhen People's Republic of China

**Keywords:** androgen receptor, flap endonuclease 1, MAPK/ERK signalling pathway, prostate cancer

## Abstract

**Purpose:**

Flap endonuclease 1 (FEN1) is highly upregulated in prostate cancer and promotes the growth of prostate cancer cells. Androgen receptor (AR) is the most critical determinant of the occurrence, progression, metastasis, and treatment of prostate cancer. However, the effect of FEN1 on docetaxel (DTX) sensitivity and the regulatory mechanisms of AR on FEN1 expression in prostate cancer need to be further studied.

**Methods:**

Bioinformatics analyses were performed using data from the Cancer Genome Atlas and the Gene Expression Omnibus. Prostate cancer cell lines 22Rv1 and LNCaP were used. FEN1 siRNA, FEN1 overexpression plasmid, and AR siRNA were transfected into cells. Biomarker expression was evaluated by immunohistochemistry and Western blotting. Apoptosis and the cell cycle were explored using flow cytometry analysis. Luciferase reporter assay was performed to verify the target relationship. Xenograft assays were conducted using 22Rv1 cells to evaluate the in vivo conclusions.

**Results:**

Overexpression of FEN1 inhibited cell apoptosis and cell cycle arrest in the S phase induced by DTX. AR knockdown enhanced DTX‐induced cell apoptosis and cell cycle arrest at the S phase in prostate cancer cells, which was attenuated by FEN1 overexpression. In vivo experiments showed that overexpression of FEN1 significantly increased tumour growth and weakened the inhibitory effect of DTX on prostate tumour growth, while AR knockdown enhance the sensitivity of DTX to prostate tumour. AR knockdown resulted in FEN1, pho‐ERK1/2, and pho‐ELK1 downregulation, and the luciferase reporter assay confirmed that ELK1 can regulate the transcription of FEN1.

**Conclusion:**

Collectively, our studies demonstrate that AR knockdown improves the DTX sensitivity of prostate cancer cells by downregulating FEN1 through the ERK/ELK1 signalling pathway.

## 1 INTRODUCTION

Prostate cancer is the second most common cancer and the fifth leading cause of cancer death in men worldwide, with nearly 1.4 million new cases and 375,000 deaths in 2020.^1^ In the United States, it is estimated that there will be 288,300 new cases of prostate cancer and 34,700 deaths in 2023.^2^ Compared with other malignant tumours, the progression of prostate cancer is slow; early stage prostate cancer can be cured by radical prostatectomy or radiotherapy. Once local tumour progression or metastasis occurs, treatment is dependent on anti‐androgen drugs.^3^

The androgen receptor (AR) is the most critical factor regulating prostate cancer occurrence, progression, and metastasis, and most treatment strategies have been developed to manipulate the AR pathway.^4^ Androgen deprivation therapy (ADT) is the standard treatment for metastatic or advanced prostate cancer.^5^ Docetaxel (DTX),^6^ abiraterone,^7^ and enzalutamide^8^ in combination with ADT are the standard treatments for patients with hormone‐sensitive prostate cancer and distant metastasis. Due to aberrant AR activation, most patients progress to metastatic castration‐resistant prostate cancer (mCRPC), which is refractory to ADT.^9^ Treatments for mCRPC include chemotherapy, new hormonal treatments (abiraterone, apalutamide and enzalutamide), radium‐223 radiotherapy^10^ and poly ADP‐ribose polymerase (PARP) inhibitors.^11^ However, drug resistance remains unavoidable and the prognosis of mCRPC is poor.^4^

Drug resistance is a significant challenge within cancer treatment. Many studies have confirmed that both ADT and chemotherapy induce DNA damage in tumour cells. Long‐term treatment leads to the enhancement of tumour cell DNA damage response (DDR), which greatly limits the anti‐tumour efficacy of ADT and chemotherapy.^12^, ^13^ The major DNA repair pathways include non‐homologous end joining for double‐strand breaks, base excision repair (BER) for single‐strand breaks, nucleotide excision repair, homologous recombination (HR) and mismatch repair.^14^

Flap endonuclease 1 (FEN1) is a structure‐specific endonuclease with flap endonuclease, 5′‐3′ exonuclease, and gap‐endonuclease activities.^15^ We have studied the role of FEN1 in drug resistance of breast cancer cells. FEN1 is critical for long‐patch BER and Okazaki fragment maturation during replication and plays an important role in stabilising telomeres as well as stalled replication fork rescue.^15^ Accumulating evidence indicates that FEN1 is necessary for tumour progression. FEN1 is highly upregulated in prostate,^16^ ovarian,^17^ breast,^18^ and lung cancers.^19^ Studies show that FEN1 blockade inhibits tumour progression and metastasis^18^ and promotes tumour cell sensitivity to anti‐tumour drugs.^17^, ^20^, ^21^ FEN1 expression increases with tumour dedifferentiation in prostate cancer, demonstrating that FEN1 expression is closely correlated with Gleason grade.^16^ Urbanucci found that nuclear FEN1 staining was stronger in CRPC than in standard PC samples, and that FEN1 can promote prostate cancer cell growth.^22^ Furthermore, AR overexpression increases FEN1 protein production in prostate cancer cells.^22^ Therefore, we speculate that AR enhances the DDR of prostate cancer cells by regulating FEN1 expression, which in turn promotes tumour growth and resistance to ADT or chemotherapy. Few studies have investigated the association between AR and FEN1 in prostate cancer.

In the classical pathway, androgen binds to the AR ligand‐binding domain (LBD) and triggers AR release from molecular chaperones; the AR LBD changes its conformation and interacts with proteins to promote nuclear translocation, thus regulating target gene expression.^23^ The AR can also enhance cell proliferation and survival through rapid, non‐genomic signalling pathways.^24^ The maintenance of this signalling pathway does not depend on AR nuclear translocation, nor does it require AR‐DNA binding. Conversely, AR activates intracellular kinase cascades primarily by binding to molecular substrates in the cytoplasm. In particular, non‐genomic AR signals are mainly dependent on MAPK/ERK activation.^25^ The MAPK/ERK signalling pathway has a key role in regulating tumour cell viability, proliferation and drug resistance, and has emerged as an important component of prostate cancer research.^26^ Based on the above, we speculate that AR rapidly regulates FEN1 expression through the MAPK/ERK signalling pathway within prostate cancer.

In this study, we confirmed that targeting AR could enhance the sensitivity of DTX to prostate cancer cells by upregulating FEN1 expression. This is likely caused via AR‐mediated activation of the MAPK/ERK signalling pathway. This study provides new insights regarding prostate cancer aetiology and therapeutics. We hypothesised that FEN1 would serve as a therapeutic target for prostate cancer.

## 2 MATERIALS AND METHODS

### 2.1 RNA sequencing analysis

Microarray prostate cancer datasets were downloaded from The Cancer Genome Atlas (TCGA) and the Gene Expression Omnibus (dataset GSE32892). Bioinformatics analyses were performed using R version 3.6.3 (The R Project for Statistical Computing). The ‘limma’ package was used to standardise mRNA sequencing results. The ‘survminer’ and ‘survival’ packages were used for survival analyses. The tumour samples in TCGA were divided into low‐expression and high‐expression groups based on the median expression of FEN1. The threshold of differentially expressed genes (DEGs) between FEN1 low‐expression and high‐expression groups was |log2FC| ≥ 1. The adjusted *p*‐value was <0.05. DAVID (https://david.ncifcrf.gov/) was used for Kyoto Encyclopaedia of Genes and Genomes (KEGG) analysis Gene set enrichment analysis (GSEA) based on WebGestalt (http://www.webgestalt.org/), with a false discovery rate (FDR) of <0.05 as the cut‐off criterion.

### 2.2 Cell culture and treatment

Human prostate cancer cell line 22Rv1 was purchased from American Type Culture Collection and cultured in RPMI 1640 with 10% foetal bovine serum (FBS). LNCaP cells were purchased from Shanghai EK‐Bioscience Co. Ltd. and maintained in RPMI 1640 supplemented with 10% FBS. Two millilitres 22Rv1 and LNCaP cells (3 × 10^5^ cells) was seeded into a six‐well culture plate for 24 h. After adding different concentrations of testosterone to 22Rv1 and LNCaP cells (10, 20 and 40 nm), the cells were cultured for another 24 h. The medium was aspirated and the cells were washed with cold PBS. Cells were collected by trypsinization in order to detect AR and FEN1 protein expression by Western blotting.

### 2.3 Cell viability assay

A total of 100 μL 22Rv1 and LNCaP cells (8 × 10^3^ cells) were seeded into a 96‐well culture plate for 24 h. After adding different concentrations of DTX, the cells were cultured for another 24 h. The medium was aspirated and the cells were washed with cold PBS; 100 μL MTT (final concentration: 5 μg/mL) was added and the cells were incubated at 37°C for 4 h. The formazan crystals were dissolved in DMSO, and the absorbance of the solution (570 nm) was determined using a multifunctional microplate reader (Nanjing Delojia Biotechnology Co. Ltd.).

### 2.4 Cell transfection

Lentiviruses were used to generate stable FEN1 knockdown/overexpression and AR knockdown cell lines. One millilitres 22Rv1 and LNCaP cells (8 × 10^4^ cells) were seeded into a 24‐well culture plate for 24 h. The medium was replaced with a serum‐free medium before viral infection. Varying volumes of virus and polybrene were added at a final concentration of 4 μg/mL. The virus titers of HBLV‐ZsGreen‐PURO and HBLV‐h‐FEN1‐3xflag‐ZsGreen‐PURO were both 2 × 10^8^ TU/mL, which was purchased from HanBio (Hanbio Biotechnology Co. Ltd.). The virus titers of pWSLV‐shFEN1‐PURO, pWSLV‐sh08‐PURO, pWSLV‐sh08‐AR1‐PURO and pWSLV‐sh08‐PURO were all 1 × 10^6^ TU/mL, which was purchased from VIEWSOLID (ViewSolid Biotechnology Co. Ltd.). The medium was replaced with fresh complete medium 6 h after transfection; the cells were collected 72 h later. Transfected cells were trypsinized and seeded into a 96‐well plate. Single colonies were transferred into six‐well plates for expansion. FEN1 or AR expression in monoclonal cells was detected via Western blotting. Clone with the highest expression were selected for further experiments.

### 2.5 Apoptosis analysis

The apoptosis of harvested cells was detected using the FITC Annexin V Apoptosis Detection kit (Pu‐nuo‐sai Life Technology Co. Ltd.). Cells were seeded into each well of a 24‐well culture plate for 24 h. The medium was aspirated and the cells were washed with cold PBS. Following digestion, the cells were centrifuged at 200 g for 5 min, the supernatant was discarded, and the cell pellet was washed with PBS. The supernatant was discarded again and the cells were resuspended in 500 μL Annexin V‐FITC binding buffer, 5 μL Annexin V‐FITC, and 7‐AAD staining solution, followed by gentle mixing. Apoptosis was analysed by flow cytometry.

### 2.6 Cell cycle assay

Cells were harvested and seeded into a 24‐well culture plate for 24 h. The medium was aspirated and the cells were washed with cold PBS. Following digestion, the cells were centrifuged at 200 g for 5 min, the supernatant was discarded, and the pellet was washed with 1 mL cold PBS. The cells were centrifuged at 200 g for 5  min and resuspended in 300 μL cold PBS; 20 μg/mL RNase A was added, followed by digestion for 30 min in a 37°C water bath. Next, 6 μL propidium iodide staining solution (30 μg/mL) was added, followed by incubation for 30 min at 4°C in the dark. The cell cycle assay was performed using flow cytometry.

### 2.7 Luciferase reporter assay

22Rv1 cells were seeded in 24 well plates (2 × 10^4^ cells/well) at 37°C with 5% CO_2_ 1 day prior to transfection. After 24 h, 1 μg of plasmid, 2 μL of X‐tremeGENE HP (Roche) and Opti‐MEM (GIBCO) were added to each well and cells were harvested 24–48 h post‐transfection; 20 μL of the cell lysate was subjected to firefly luciferase assay (40 μL of Luciferase Assay Reagent; Promega). Stop & Glo® Reagent (40 μL) was added, and the second luminescence measurement was taken. Renilla luminescence was measured using a Dual‐Luciferase® Reporter Assay System (E1910, Promega) on a microplate reader (BioTek).

### 2.8 In vivo mouse model

50 BALB/c male nude mice (4–5 weeks old) were housed in a standard animal room; the mice were free to eat and drink and were kept at an indoor temperature of 20–26°C. The mice were randomly divided into experimental group and control group. 22Rv1 cells (2 × 10^6^) were subcutaneously injected into the right axillary fossa. Tumour volume (mm^3^) was determined by measurement with Vernier callipers and the following formula: volume = length × width × width × π/6. Treatment was initiated when tumours reached 150–200 mm^3^ in volume. Mice were injected intraperitoneally with DTX (15 mg/kg) or PBS (0.5% DMSO) once a week for 4 weeks. Body weight was measured every 3 days. One week after injection, the mice were euthanized, and the tumours were dissected, weighed, and fixed in 10% formalin. Half the tumour was fixed in 4% PFA and embedded in paraffin. The other half was flash frozen into liquid nitrogen and enzymatically digested into single‐cell suspensions. All animal ethics and procedures were approved by the Medical Animal Care & Welfare Committee at Shenzhen University Health Science Center.

### 2.9 Immunohistochemistry analysis

Paraffin sections were baked in an oven at 65°C for 2 h and deparaffinized with xylene (2 × 10 min), 100% ethanol (5 min), 95% ethanol (5 min), 90% ethanol (5 min), 80% ethanol (5 min), 70% ethanol (5 min) and distilled water (5 min). Endogenous peroxidase activity was blocked with 3% H_2_O_2_ for 15 min. The sections were washed three times with PBS (5 min). Antigen retrieval was performed by microwaving sections in 0.01 M sodium citrate buffer (pH 6.0) for 15 min and washed three times with PBS. The sections were blocked for 90 min at room temperature in 10% horse serum blocking solution and incubated with diluted primary antibody (Ki67, 1:200; FEN1, 1:200; pho‐ERK1/2, 1:100; pho‐ELK1, 1:100; Cell Signaling Technology and Abcam) at 4°C overnight, followed by three 5 min washes with PBS, secondary antibody incubation (anti‐mouse immunoglobulin G [IgG], anti‐rabbit IgG, HRP‐linked antibody, cell signalling technology at 1:2000 dilution). The tissues were coloured with yellow DAB chromogenic reagent and the degree of coloration was observed under a microscope.

### 2.10 Western blotting

The harvested cells were lysed in 30 μL RIPA cell lysis buffer (Beyotime), incubated on ice for 30 min, centrifuged at 12,000 rpm for 20 min at 4°C, and the supernatant was used as the cell lysate. The protein concentration of the lysate was determined using the Pierce BCA Protein Assay Kit (Beyotime). The lysate was electrophoresed on an 8% SDS polyacrylamide gel and transferred to a PVDF membrane (Beyotime), which was washed in TBST, blocked with 5% BSA for 1 h, incubated in primary antibody solution at 4°C overnight, washed with TBST three times (10 min) and incubated with secondary antibody for 1 h. Membranes were washed with TBST three times (10 min). The primary antibodies included AR (dilution 1:1000), FEN1 (dilution 1:2000), pro‐ERK1/2 (dilution 1:2000), ERK1/2 (dilution 1:1000), pho‐ELK1 (dilution 1:1000), β‐actin (dilution 1:1000) and GAPDH (dilution 1:1000) (Cell Signaling Technology and Abcam). Chemiluminescence was recorded using a ChemiDoc Imaging System (Bio‐Rad). Quantitative analysis was completed using ImageJ software (National Institutes of Health).

### 2.11 Statistical analysis

Images were analysed and semi‐quantified using Image Pro Plus software (Media Cybernetics). Flow J (version 10) software was used to analyse flow cytometry data. Experimental data were statistically analysed using GraphPad Prism 8.0 and expressed as mean ± standard deviations (SD). Multi‐group comparisons were performed using one‐way analysis of variance (one‐way ANOVA) with Dunnett's post hoc test for normally distributed data. Two‐way ANOVA with Tukey's post hoc test was used for ≥2 groups with two variables. Statistical significance was set at *p* < 0.05.

## 3 RESULTS

### 3.1 The FEN1 expression in prostate cancer and its relationship with androgen receptor

**FIGURE 1:**
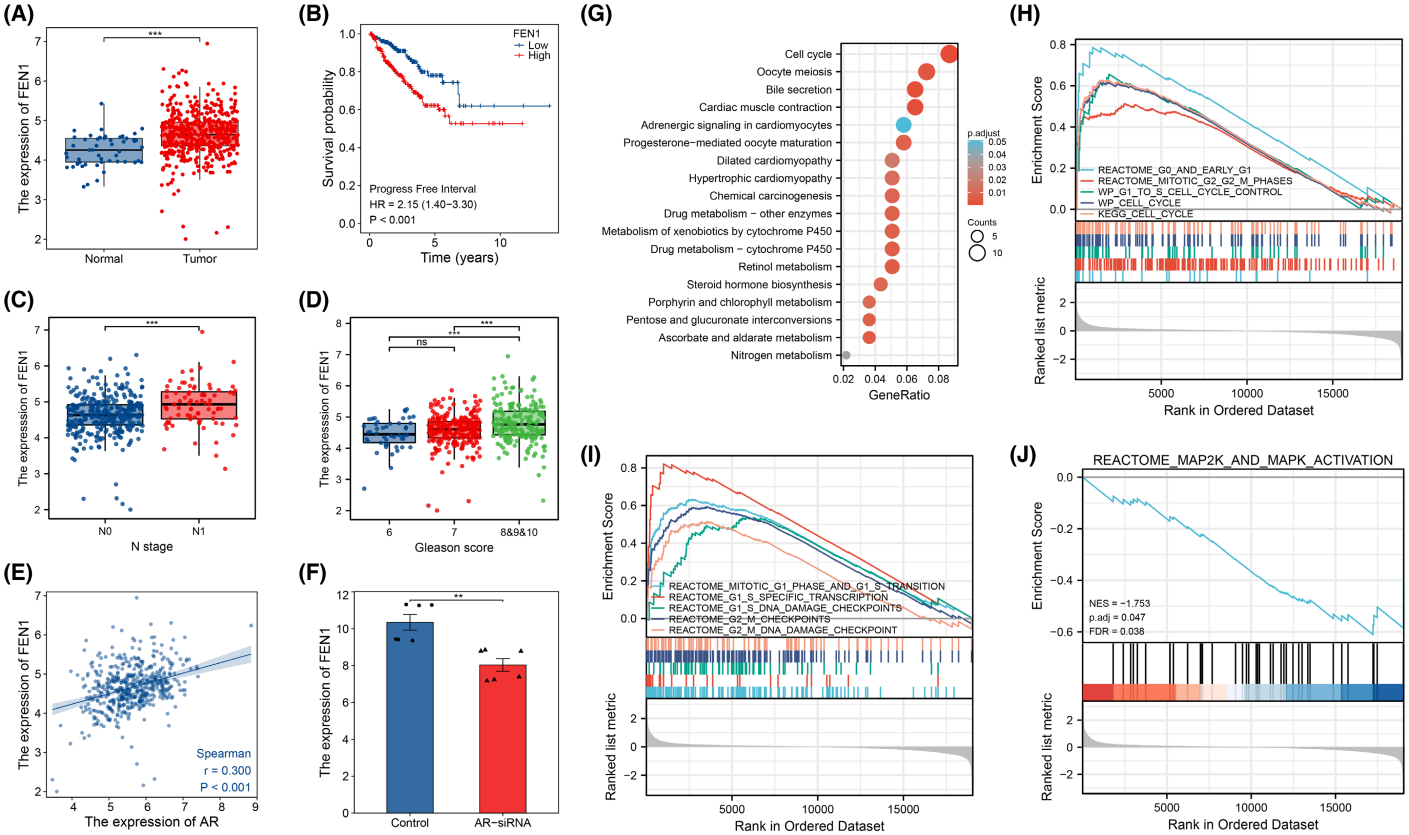
DEGs analyses of FEN1 and AR in public databases (TCGA dataset and GEO dataset GSE32892). (A) Boxplot showing FEN1 expression in normal and prostate cancer tissues. (B) Kaplan–Meier analysis of progress‐free survival, comparing low and high FEN1 expression. (C) Boxplot showing FEN1 expression in prostate cancer tissues with different N stage. (D) Boxplot showing FEN1 expression in prostate cancer tissues with different Gleason score. (E) The correlation analyses evaluating FEN1 expression and AR in prostate cancer tissues was performed within the TCGA dataset. Spearman's correlation test was used to analyse the correlation between FEN1 and AR expression. (F) The DEG analyses was performed with the GSE32892 dataset. Barplot showing FEN1 expression in VCaP cells between control and AR‐siRNA group. (G) The Kyoto Encyclopedia of Genes and Genomes (KEGG) enrichment analyses of 367 DEGs between the FEN1 low‐expression and high‐expression groups within the TCGA dataset. The top 18 most significantly enriched KEGG terms are shown. (H–J) The GSEA analyses showed that FEN1 was involved in cell cycle activity and MAPK signalling pathway in prostate cancer. Data are presented as mean ± SD values. **p < 0.01, ***p < 0.001, ns, no significance. NES, normalise enrichment score, *p*.adjust, adjusted *p* value, FDR, false discovery rate.

To clarify the expression and role of FEN1 in prostate cancer, we conducted bioinformatics analyses of public databases (TCGA and GSE32892). FEN1 expression in prostate cancer tissues was statistically significantly higher than that in normal tissues (Figure 1A). Low expression of FEN1 in prostate cancer indicated better progression‐free survival (HR = 2.15, *p* < 0.001) (Figure 1B). Further analyses of FEN1 expression and clinical characteristics of prostate cancer demonstrated that higher FEN1 expression was closely related to a higher N stage and Gleason score (Table 1, Figure 1C,D). These results suggest that FEN1 expression is upregulated in prostate cancer and is associated with a poor prognosis. We also found FEN1 expression was positively correlated with AR expression in prostate cancer (*r* = 0.3, *p* < 0.001) (Figure 1E). When AR was knocked down in prostate cancer cells, FEN1 expression was also downregulated (Figure 1F). These results strongly suggest that AR plays a biological role partly by regulating FEN1 expression.

**TABLE 1:** Clinical characteristics of 499 samples according to FEN1 expression.

Characteristic	Low expression of FEN1	High expression of FEN1	*p*
*n*	249	250	
Age, mean ± SD	60.99 ± 6.86	61.07 ± 6.79	0.896
T stage, *n* (%)			0.165
T2	104 (21.1%)	85 (17.3%)	
T3	138 (28%)	154 (31.3%)	
T4	4 (0.8%)	7 (1.4%)	
N stage, *n* (%)			**0.002**
N0	179 (42%)	168 (39.4%)	
N1	25 (5.9%)	54 (12.7%)	
M stage, *n* (%)			1.000
M0	225 (49.1%)	230 (50.2%)	
M1	1 (0.2%)	2 (0.4%)	
Gleason score, *n* (%)			**0.005**
6	25 (5%)	21 (4.2%)	
7	142 (28.5%)	105 (21%)	
8	27 (5.4%)	37 (7.4%)	
9	54 (10.8%)	84 (16.8%)	
10	1 (0.2%)	3 (0.6%)	
OS event, *n* (%)			0.106
Alive	247 (49.5%)	242 (48.5%)	
Dead	2 (0.4%)	8 (1.6%)	
DSS event, *n* (%)			0.216
Alive	248 (49.9%)	244 (49.1%)	
Dead	1 (0.2%)	4 (0.8%)	
PFI event, *n* (%)			**< 0.001**
Alive	217 (43.5%)	188 (37.7%)	
Dead	32 (6.4%)	62 (12.4%)	

Bold value indicates *p* < 0.05.

The enrichment analysis of KEGG was carried out to reveal the biological pathways. The enriched signalling pathways included the hsa04110 Cell cycle, hsa04114 oocyte meiosis, hsa04976 bile secretion, hsa04260 cardiac muscle contraction and others (Figure 1G). Furthermore, a GSEA study was performed to investigate the change in enriched signalling pathways. We found that cell cycle‐related pathways and MAPK pathway were significantly upregulated (Figure 1H–J). Taken together, these findings indicated that FEN1 was related to cell cycle activity and MAPK pathway in prostate cancer.

### 3.2 Testosterone and docetaxel influence the protein level of AR and FEN1 in prostate cancer cells

To determine the appropriate concentrations of testosterone and DTX in subsequent cell experiments, two prostate cancer cell lines (22Rv1 and LNCaP) were used for concentration gradient experiments. By adding different concentrations of DTX (0.25, 0.5, 1, 2, 4, 6, 8 and 10 nM), we found that 22Rv1 cells were more sensitive to DTX. Furthermore, we calculated the IC_50_ of DTX in 22Rv1 and LNCaP cells using different concentrations of DTX (0.03, 0.1, 0.3, 1, 3, 10 and 30 nM). Cytotoxicity results indicated that the IC_50_ of DTX was 16.19 ± 0.20 nm and 20.10 ± 4.80 nm for 22Rv1 and LNCaP cells, respectively (Figure S1A).

Next, we examined the effects of DTX on AR and FEN1 expression. In 22Rv1 cells, DTX statistically significantly decreased AR expression at 16 nm (*p* < 0.05), and statistically significantly decreased FEN1 expression at 8 and 16 nm (*p* < 0.01). In LNCaP cells, AR expression was statistically significantly decreased by DTX treatment at 20 nm (*p* < 0.01), while FEN1 expression was statistically significantly decreased at 10 and 20 nm (*p* < 0.01, Figure S1B).

We further found that testosterone increased AR and FEN1 protein levels in 22Rv1 cells in a dose‐dependent manner. Similar results were observed in LNCaP cells (Figure S1C). In 22Rv1 cells, testosterone statistically significantly increased AR and FEN1 expression at 20 nm (*p* < 0.05) and 40 nm (*p* < 0.01). In LNCaP cells, testosterone statistically significantly increased AR expression at 20 and 40 nm (*p* < 0.001) and statistically significantly increased FEN1 expression at 10, 20, and 40 nm (*p* < 0.05).

Therefore, the concentration of testosterone for subsequent experiments was determined to be 20 nm. The concentration of DTX was determined as 16 and 20 nm for 22Rv1 and LNCaP cells, respectively. A previous study showed that AR overexpression increased FEN1 protein levels in prostate cancer cells.^22^ We speculated that the decrease in FEN1 protein level caused by DTX was partly due to the decrease in AR protein levels.

### 3.3 FEN1 reduces the DTX sensitivity of prostate cancer cells

**FIGURE 2:**
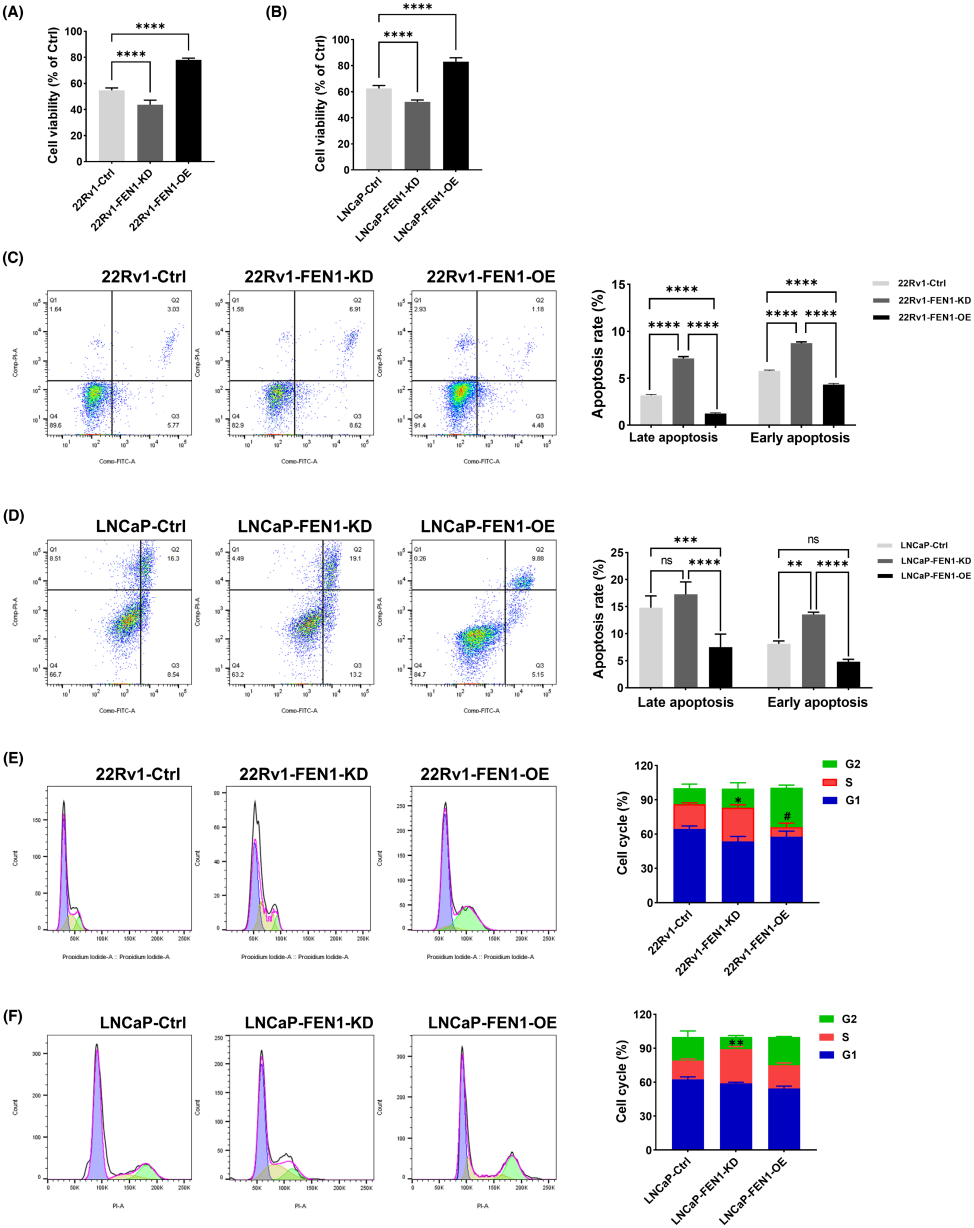
Effects of FEN1 overexpression or konckdown on cell viability, cell apoptosis, and cell cycle in 22Rv1 and LNCaP cells. (A) Effects of FEN1 expression on cell viability in 22Rv1 cells treated with docetaxel (DTX) (*n* = 9 per group). (B) Effects of FEN1 expression on cell viability in LNCaP cells treated with DTX (*n* = 9 per group).(C) Effects of FEN1 expression on apoptosis in 22Rv1 cells treated with DTX (*n* = 3 per group). (D) Effects of FEN1 expression on apoptosis in LNCaP cells treated with DTX (*n* = 3 per group). (E) Effects of FEN1 expression on cell cycle in 22Rv1 cells treated with DTX (*n* = 3 per group). *p < 0.05, 22Rv1‐FEN1‐KD versus 22Rv1‐Ctrl; ^#^ *p* < 0.05, 22Rv1‐FEN1‐OE versus 22Rv1‐Ctrl.(F) Effects of FEN1 expression on cell cycle in LNCaP cells treated with DTX (*n* = 3 per group). The data are presented as the mean ± SD values, and the error bars represent data from triplicate or more biological experiments. **p < 0.01, LNCaP‐FEN1‐KD versus LNCaP‐Ctrl. *p < 0.05, **p < 0.01, ***p < 0.001, ****p < 0.0001, ns, no significance. The concentration of DTX was determined as 16 and 20 nm for 22Rv1 and LNCaP cells. The cells were collected after 48 h treatment. The cell viability was obtained by MTT assay.

To determine the biological function of FEN1 in prostate cancer, we knocked down and overexpressed FEN1 in prostate cancer cells (Figures S2 and S3). Therefore, we divided 22Rv1 cells into the following groups: 22Rv1‐Ctrl, 22Rv1‐FEN1‐KD (knockdown) group and 22Rv1‐FEN1‐OE (overexpression) groups. LNCaP cells were similarly grouped. All groups were treated with DTX. The viability of 22Rv1 and LNCaP cells decreased substantially following DTX treatment (Figure S4A,D). After FEN1 was knockdowned, the cell viability decreased as compared with the 22Rv1‐Ctrl (43.67 ± 3.53 vs. 54.7 ± 1.89, *p* < 0.0001) and LNCaP‐Ctrl groups (52.29 ± 1.41 vs. 62.64 ± 2.18, *p* < 0.0001) (Figure 2A,B). Conversely, FEN1 overexpression resulted in a statistically significant increase in tumour cell viability compared with the 22Rv1‐Ctrl (78.12 ± 1.34 vs. 54.7 ± 1.89, *p* < 0.0001) and LNCaP‐Ctrl groups (83.15 ± 2.99 vs. 62.64 ± 2.18, *p* < 0.0001) (Figure 2A,B).

Flow cytometric analysis demonstrated results similar to those of apoptosis detection. There were no statistically significant differences in the apoptotic rate in early and late apoptosis among cell groups without DTX (*p* > 0.05, Figure S4B,E). This demonstrated that, in the absence of DTX, FEN1 expression had no effect on cells' apoptotic rates. After the cells were treated with DTX, the apoptotic rate increased, especially during late apoptosis (Figure S4B,E). During early and late apoptosis, FEN1 knockdown increased the apoptotic rate as compared with the control groups in both 22Rv1 and LNCaP cells (*p* < 0.0001, Figure 2C,D). In addition, FEN1 overexpression decreased the apoptosis rate as compared with the control groups both in early and late apoptosis (all *p* < 0.0001, Figure 2C,D).

We also evaluated 22Rv1 cells and LNCaP cells to test whether FEN1 expression is linked to cell cycle progression in prostate cancer cells. First, DTX treatment led to an increase in the proportion of S phase cells in each group (Figure S4C,F). The proportion of S phase cells in the 22Rv1‐FEN1‐KD group was the highest (29.63 ± 2.11%). The proportion of S phase cells in 22Rv1‐FEN1‐OE group (8.32 ± 2.95%) was statistically significantly lower than that in 22Rv1‐Ctrl and 22Rv1‐FEN1‐KD groups (both *p* < 0.05, Figure 2C). In LNCaP cells, FEN1 knockdown induced more S phase cells (30.27 ± 1.93%) compared to the LNCaP‐Ctrl group (*p* < 0.01, Figure 2E). In addition, FEN1 overexpression reduced the proportion of S phase cells (20.47 ± 1.65%) compared to the LNCaP‐FEN1‐KD group.The results indicated that DTX induced S‐phase arrest in prostate cancer cells, which could be partially reversed by FEN1 overexpression.

These results suggest that FEN1 expression has a strong impact on DTX sensitivity in prostate cancer cells. High FEN1 expression levels correlated with lower DTX sensitivity, whereas low FEN1 expression levels correlated with higher sensitivity to DTX in prostate cancer cells.

### 3.4 FEN1 eliminates the DTX sensitivity of prostate cancer cells enhanced by AR knockdown

**FIGURE 3:**
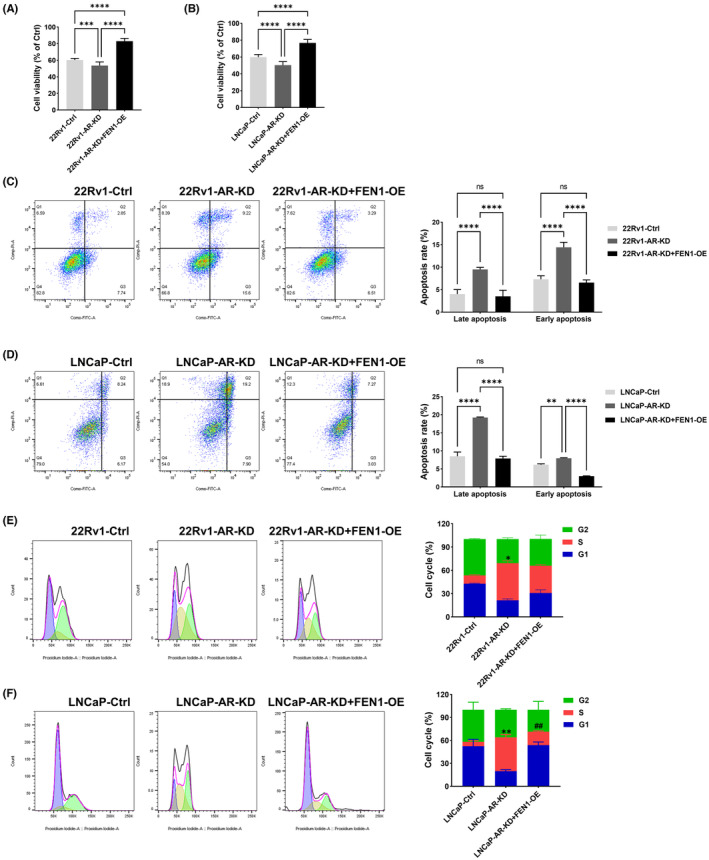
Effects of AR knockdown and FEN1 overexpression on cell viability, cell apoptosis, and cell cycle in 22Rv1 and LNCaP cells treated with DTX. (A) Effects of AR knockdown and FEN1 overexpression on cell viability in 22Rv1 cells treated with DTX (*n* = 9 per group). (B) Effects of AR knockdown and FEN1 overexpression on cell viability in LNCaP cells treated with DTX (*n* = 9 per group). (C) Effects of AR knockdown and FEN1 overexpression on apoptosis in 22Rv1 cells treated with DTX (*n* = 3 per group). (D) Effects of AR knockdown and FEN1 overexpression on apoptosis in LNCaP cells treated with DTX (*n* = 3 per group). (E) Effects of AR knockdown and FEN1 overexpression on cell cycle in 22Rv1 cells treated with DTX (*n* = 3 per group). *p < 0.05, 22Rv1‐FEN1‐KD versus 22Rv1‐Ctrl. (F) Effects of AR knockdown and FEN1 overexpression on LNCaP cells cycle treated with DTX (*n* = 3 per group). Data are presented as the mean ± SD values, and the error bars represent data from triplicate or more biological experiments. **p < 0.01, LNCaP‐AR‐KD vs. LNCaP‐Ctrl; ^##^ *p* < 0.01, LNCaP‐AR‐KD+FEN1‐OE versus LNCaP‐AR‐KD. *p < 0.05, **p < 0.01, ***p < 0.001, ****p < 0.0001, ns, no significance.

AR plays an important role in the tumorigenesis, progression and metastasis of prostate cancer, and is an important target for prostate cancer treatment.^27^ Thus we explored whether AR knockdown promoted toxicity effects of DTX on prostate cancer cell growth. As expected, AR protein levels showed a statistically significant decrease in prostate cancer cells after AR was knocked down as compared to cells in the control group (Figure S2 and S3). We divided 22Rv1 cells into the following groups: 22Rv1‐Ctrl, 22Rv1‐AR‐KD and 22Rv1‐AR‐KD+FEN1‐OE groups. LNCaP cells were similarly grouped. All groups were treated with DTX and 20 nM testosterone. Consistent with previous experimental results, DTX treatment statistically significantly reduced the cell viability within 22Rv1 and LNCaP cells (Figure S5). After AR was knocked down, the cell viability decreased as compared with the 22Rv1‐Ctrl (53.65 ± 4.35 vs. 60.29 ± 1.94, *p* < 0.001) and LNCaP‐Ctrl groups (50.37 ± 4.15 vs. 59.85 ± 3.11, *p* < 0.0001) (Figure 3A,B). The results of apoptosis experiments also showed that the 22Rv1‐AR‐KD group had a statistically significantly higher apoptotic rate than the 22Rv1‐Ctrl group both in late apoptosis (all *p* < 0.0001) and early apoptosis (all *p* < 0.0001, Figure 3C). The same result was confirmed in LNCaP cells (Figure 3D).

Interestingly, when FEN1 was overexpressed in AR‐knockdowned cells, cell viability increased statistically significantly compared as with the 22Rv1‐AR‐KD (83.04 ± 3.33 vs. 53.65 ± 4.35, *p* < 0.0001) or LNCaP‐AR‐KD groups (76.81 ± 4.04 vs. 50.37 ± 4.15, *p* < 0.0001) (Figure 3A,B). Furthermore, stable FEN1 overexpression reduced the cell apoptotic rate caused by AR knockdown in 22Rv1 and LNCaP cells (both *p* < 0.0001) (Figure 3C,D). These results suggest that AR knockdown improved the DTX sensitivity of prostate cancer cells, while FEN1 eliminated DTX sensitivity in prostate cancer cells enhanced by AR knockdown.

Furthermore, AR knockdown combined with DTX treatment induced the most S‐phase arrest in both 22Rv1 and LNCaP cells (51.07% ± 5.04, 44.27% ± 3.03) as compared with the control groups. Although the proportion of S phase cells also increased in the AR‐KD+FEN1‐OE groups (33.50% ± 2.43, 17.61% ± 0.88), the increases were less than in the AR‐KD group (Figure 3E,F). The results of these cell cycle experiments demonstrate that S‐phase arrest in prostate cancer cells induced by DTX was enhanced by AR knockdown. Moreover, this phenomenon was partially eliminated by FEN1 overexpression.

### 3.5 FEN1 reduces DTX chemosensitivity of prostate cancer in vivo

**FIGURE 4:**
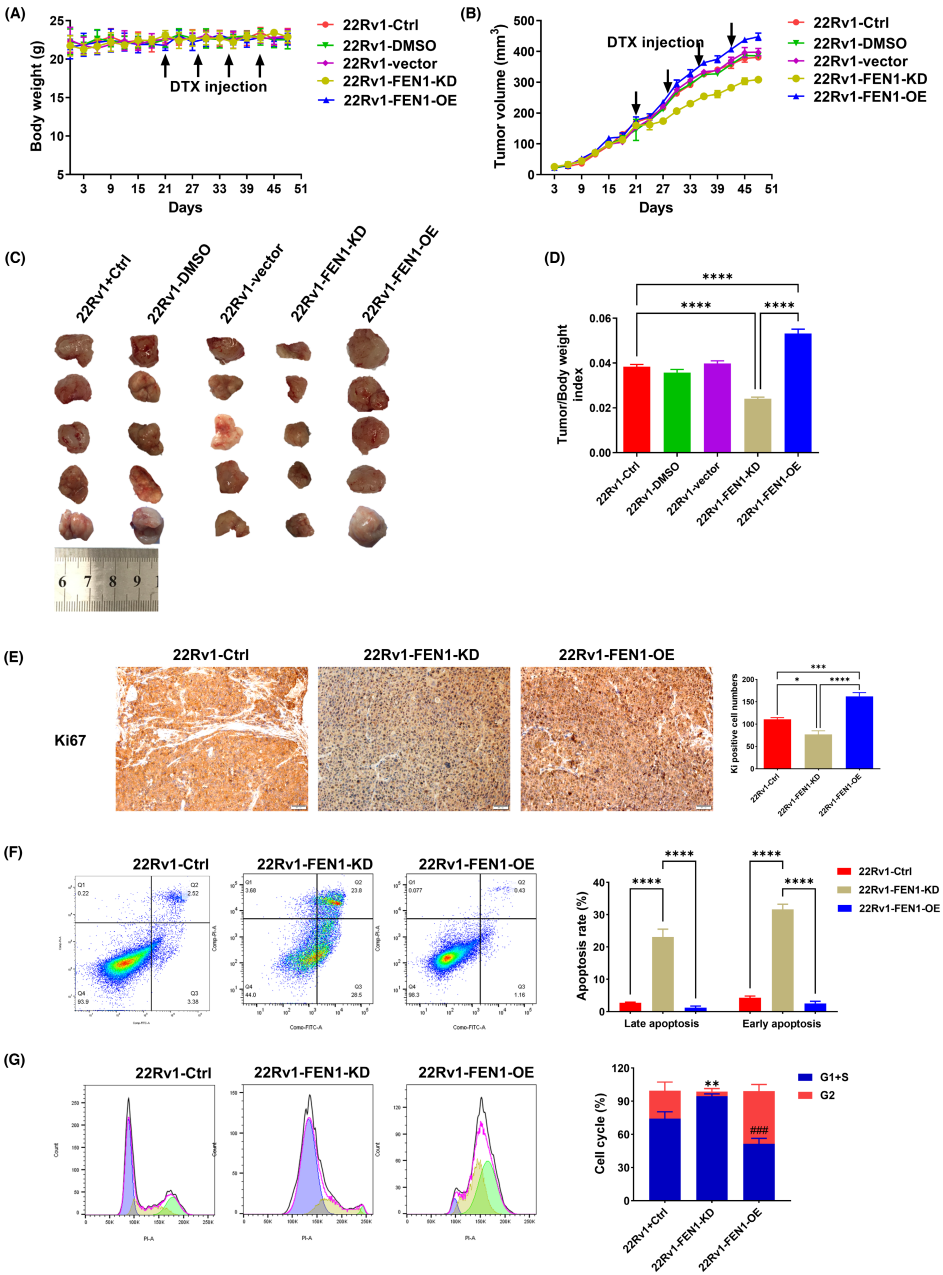
Effects of FEN1 overexpression or knockdown on sensitivity to DTX in a prostate cancer model. (A) Body weight in nude mice after DTX injection. (B) Tumour volume in nude mice after DTX injection. (C) Images of xenograft tumour. (D) Tumour/body weight indexes in nude mice. (E) Typical immunohistochemical photographs and semi‐quantitative analyses of Ki67 expression in tumour tissues of nude mice. (F) Flow cytometry showed the effects of FEN1 knockdown and overexpression on tumour cell apoptosis in cells treated with DTX in vivo. (G) Flow cytometry showed the effects of FEN1 knockdown and overexpression on tumour cells cycle treated with DTX in vivo. Data are presented as the mean ± SD values, and the error bars represent data from quintuplicate biological experiments. **p < 0.01, 22Rv1‐FEN1‐KD versus 22Rv1‐Ctrl; ^###^ *p* < 0.001, 22Rv1‐FEN1‐OE versus 22Rv1‐Ctrl. *p < 0.05, **p < 0.01, ***p < 0.001, ****p < 0.0001.

To determine whether FEN1 expression influences tumour growth in vivo, 22Rv1 cells with normal FEN1 expression, knockdown and overexpression were subcutaneously injected into mice treated with DTX. The nude mice were divided into the following five groups: 22Rv1‐Ctrl, 22Rv1‐DMSO, 22Rv1‐vector, 22Rv1‐FEN1‐KD and 22Rv1‐FEN1‐OE. Tumour growth and mouse weight were monitored every 3 days. There was no statistically significant difference in body weight for nude mice before and after DTX injection (Figure 4A). As shown in Figure 4B, compared with the 22Rv1‐control, 22Rv1‐DMSO and 22Rv1‐vector groups, the tumour volume in the 22Rv1‐FEN1‐KD group decreased statistically significantly (all *p* < 0.001). Compared with the 22Rv1‐FEN1‐KD group, the tumour volume of the 22Rv1‐FEN1‐OE group increased statistically significantly (*p* < 0.001). At the end of the experiment, the tumour was dissected and weighed (Figure 4C) and the tumour index was calculated (Figure 4D). Compared with the 22Rv1‐Ctrl group, the tumour/body weight index of nude mice in the 22Rv1‐FEN1‐KD group decreased statistically significantly (*p* < 0.0001), while that of the 22Rv1‐FEN1‐OE group increased statistically significantly (all *p* < 0.0001). These results indicated that FEN1 knockdown statistically significantly reduced tumour growth, whereas FEN1 overexpression statistically significantly increased tumour volume.

Considering the importance of FEN1 in tumour growth, we assessed the effects of FEN1 on Ki67 expression by immunohistochemistry (IHC). Tumour tissues with FEN1 knockdown showed lower levels of Ki67, while Ki67 expression was statistically significantly increased in tumour tissues with FEN1 overexpression as compared with other mice in the 22Rv1‐Ctrl group (Figure 4E). Flow cytometry results also showed FEN1 knockdown led to increased apoptosis and G1/S phase arrest in tumour cells treated with DTX in vivo, whereas FEN1 overexpression achieved the opposite results (Figure 4F,G). The above findings suggest that FEN1 promotes the proliferation and inhibits the cell cycle arrest of prostate cancer cells in vivo.

### 3.6 FEN1 reverses DTX chemosensitivity of prostate cancer enhanced by AR knockdown in vivo

**FIGURE 5:**
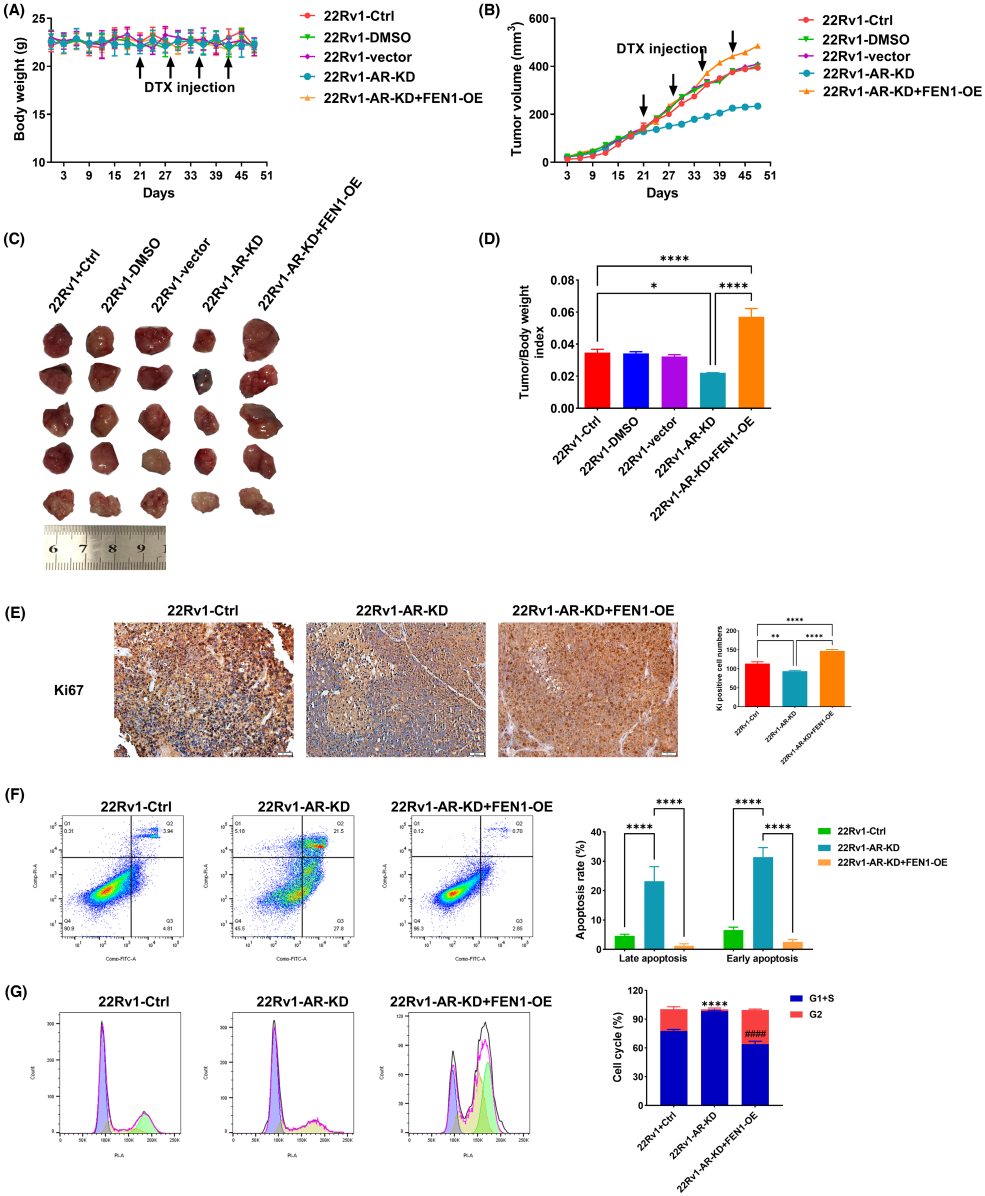
Effect of AR knockdown and FEN1 overexpression on DTX sensitivity in a prostate cancer model. (A) Body weight of nude mice after DTX injection. (B) Tumour volume of nude mice after DTX injection. (C) Images of xenograft tumour. (D) Tumour/body weight indexes in nude mice. (E) Typical immunohistochemical photographs and semi‐quantitative analyses of Ki67 expression in tumour tissues in nude mice. (F) Flow cytometry showed the effects of FEN1 overexpression and AR knockdown on tumour cell apoptosis in cells treated with DTX in vivo. (G) Flow cytometry showed the effects of FEN1 overexpression and AR knockdown on tumour cells cycle treated with DTX in vivo. Data are presented as the mean ± SD values, and the error bars represent data from quintuplicate biological experiments. ****p < 0.0001, 22Rv1‐AR‐KD versus 22Rv1‐Ctrl; ^####^ *p* < 0.0001, 22Rv1‐AR‐KD+FEN1‐OE versus 22Rv1‐AR‐KD. *p < 0.05, **p < 0.01, ****p < 0.0001.

AR knockdown enhances the sensitivity of prostate cells to DTX in vitro. Thus, we evaluated the effects of AR knockdown in vivo. Similarly, we transfected 22Rv1 cells with normal AR expression, AR knockdown and AR knockdown+FEN1 overexpression into mice treated with DTX. Similarly, nude mice were divided into the following five groups: 22Rv1‐Ctrl, 22Rv1‐DMSO, 22Rv1‐vector, 22Rv1‐AR‐KD and 22Rv1‐AR‐KD+FEN1‐OE. No statistically significant differences were found in the body weights of nude mice before and after DTX injection (Figure 5A). As expected, tumours with AR knockdown showed decreased tumour growth as compared with the 22Rv1‐Ctrl group (*p* < 0.05, Figure 5B–D). In addition, tumour volume and tumour/body weight index were statistically significantly increased in the 22Rv1‐si‐AR+FEN1‐OE group as compared with the 22Rv1‐control and 22Rv1‐AR‐KD groups (both *p* < 0.0001, Figure 5B–D). This indicated that AR knockdown further enhanced the inhibitory effect of DTX on prostate tumour growth, while FEN1 overexpression weakened this enhancement.

We found that AR knockdown led to downregulation of Ki67 expression in tumour tissues. However, similar to the results for tumour volume, FEN1 overexpression reversed the decreased expression of Ki67caused by AR knockdown (Figure 5E). In addition, flow cytometry results demonstrated that AR knockdown induced increased apoptosis and G1/S phase arrest in tumour cells treated with DTX in vivo.Conversely, FEN1 overexpression reversed apoptosis and G1/S phase arrest induced by DTX and AR knockdown (Figure 5F,G).

Together, these results suggest that AR knockdown inhibits prostate cancer cell proliferation, indicating that AR knockdown could enhance the sensitivity of prostate cancer cells to DTX. Moreover, FEN1 reversed the chemosensitivity enhanced by AR knockdown to DTX treatment in vivo.

### 3.7 AR knockdown inhibits the expression of FEN1 and MAPK signal pathways

**FIGURE 6:**
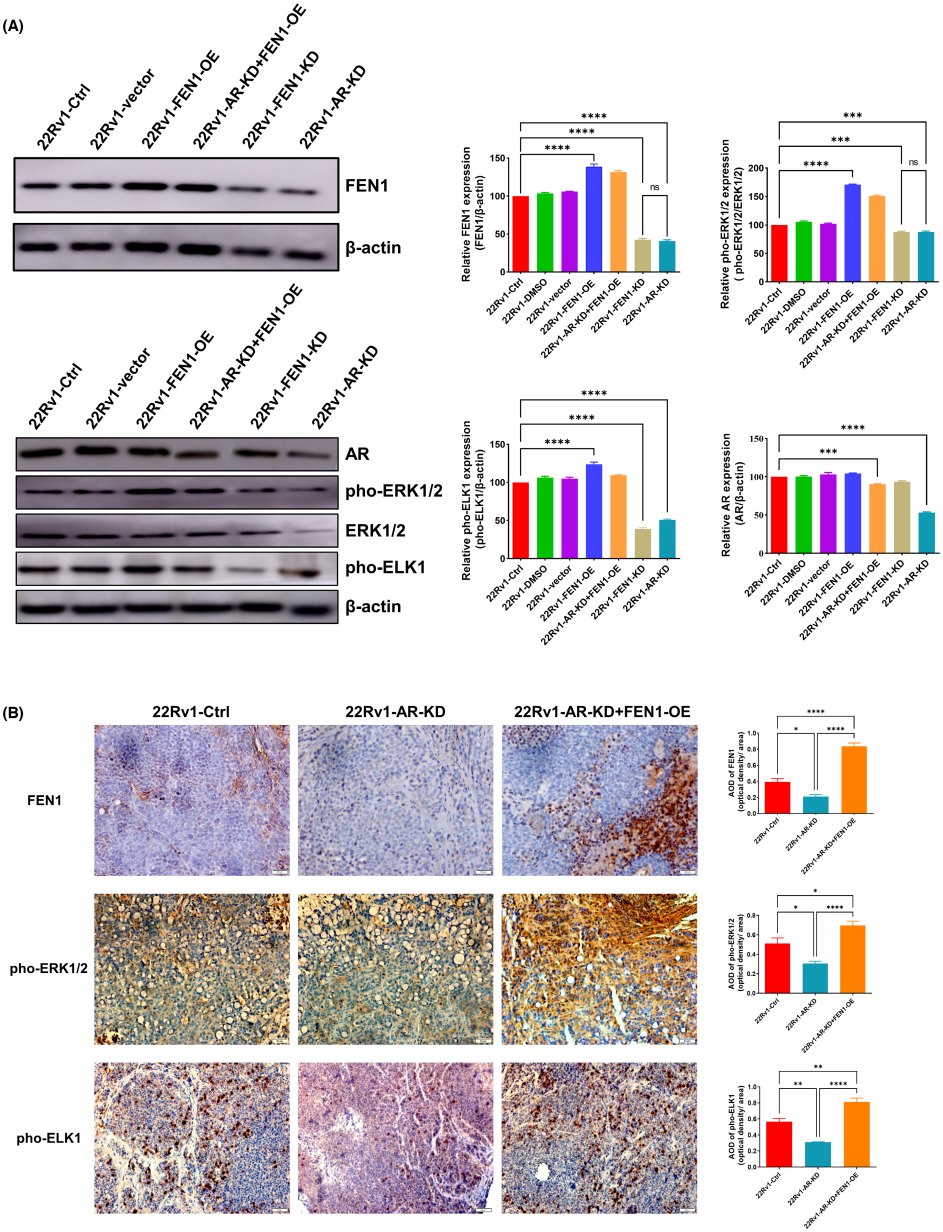
AR knockdown decreased the expression of AR, FEN1, pho‐ERK1/2 and pho‐ELK1 in tumour tissues. (A) The protein expression of AR, FEN1, pho‐ERK1/2 and pho‐ELK1 in tumour tissues of nude mice. (B) Typical immunohistochemical photographs and semi‐quantitative analyses of FEN1, pho‐ERK1/2 and pho‐ELK1 expression in tumour tissues of nude mice. Data are presented as the mean ± SD values, and the error bars represent data from triplicate biological experiments. *p < 0.05, **p < 0.01, ***p < 0.001, ****p < 0.0001, ns, no significance.

We further studied the effect of AR knockdown on AR, FEN1, pho‐ERK1/2 and pho‐ELK1 expression in tumour tissues by Western blot. AR knockdown statistically significantly decreased FEN1, pho‐ERK1/2 and pho‐ELK1 expression in tumour tissues of nude mice as compared with the 22Rv1‐control group (all *p* < 0.001; Figure 6A). Similar results were obtained by knocking down FEN1 (all *p* < 0.001), while FEN1 overexpression resulted in the opposite findings (all *p* < 0.0001). In addition, FEN1 overexpression reversed the downregulation of FEN1, pho‐ERK1/2 and pho‐ELK1 induced by AR knockdown (all *p* < 0.0001). Likewise, the IHC results demonstrated similar results to Western blot (Figure 6B). We confirmed the regulatory effect of AR on FEN1, pho‐ERK1/2 and pho‐ELK1 in prostate cancer cells. However, the role of the MAPK/ERK signalling pathway in the regulation of FEN1 by AR remains to be elucidated.

### 3.8 AR knockdown inhibits the expression of FEN1 by regulating ERK/ELK1 signalling pathway

**FIGURE 7:**
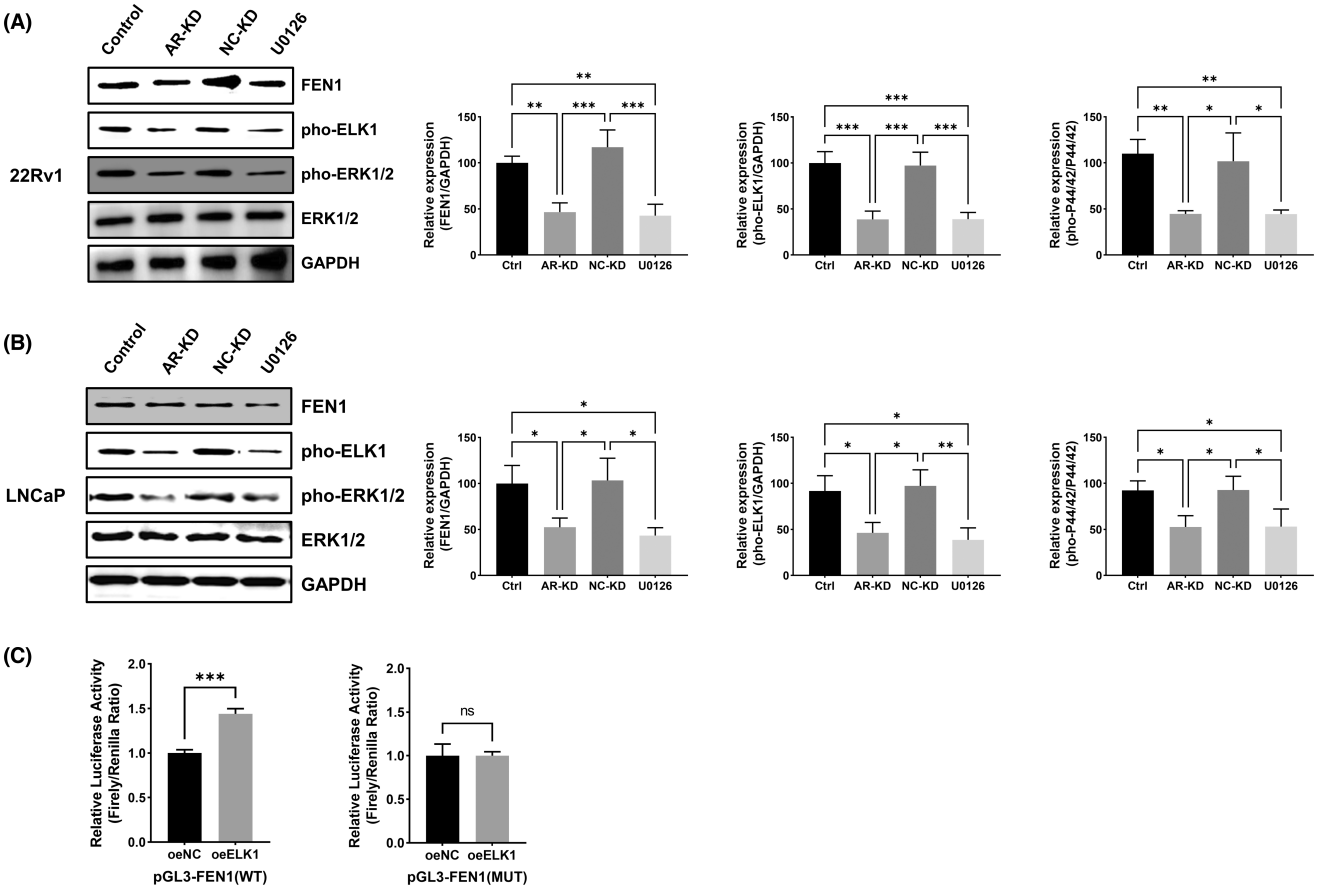
Regulatory associations among AR, ELK, and FEN1 in prostate cancer cells. (A) Western blot results showing the effects of AR knockdown and U0126 on ELK1 and FEN1 expression in 22Rv1 cells. (B) Western blot results showing the effects of AR knockdown and U0126 on ELK1 and FEN1 expression in LNCaP cells. (C) Luciferase reporter assay results showing that ELK1 could regulate the transcription of FEN1. Data are presented as the mean ± SD values, and the error bars represent data from triplicate biological experiments. *p < 0.05, **p < 0.01, ***p < 0.001; ns, no significance.

To further verify the effect of the AR and MAPK/ERK signalling pathways on FEN1 expression in prostate cancer, AR was knocked down and the MAPK/ERK kinase (MEK) inhibitor U0126 was implemented in 22Rv1 and LNCaP cells. We divided 22Rv1 cells into the following groups: 22Rv1‐Ctrl, 22Rv1‐AR‐KD, 22Rv1‐NC‐KD and 22Rv1+U0126. LNCaP cells were similarly grouped. As shown in Figure 7A, FEN1 expression was statistically significantly downregulated in the 22Rv1‐AR‐KD group as compared with the 22Rv1‐Ctrl and 22Rv1‐NC‐KD groups (both *p* < 0.01); this confirmed that AR regulates FEN1 expression. AR knockdown also statistically significantly downregulated pho‐ERK1/2 and pho‐ELK1 expression (all *p* < 0.05) and U0126 treatment achieved the same effect as AR knockdown (all *p* < 0.05). We obtained similar results in LNCaP cells (Figure 7B). These results preliminarily proved that AR could regulate FEN1 expression in prostate cancer cells through the ERK/ELK1 signalling pathway. Since ETS transcription factor 1 (ELK1) is a transcriptional factor, we investigated whether ELK1 could directly activate FEN1 transcription.

We used JASPAR and UCSC tools for the transcription factor binding site analysis. We found that ELK1 has five binding sites for the FEN1 promoter, and the site with the strongest score was selected. The pGL3 luciferase reporter plasmids containing the 3′‐UTR sequences of wild‐type FNE1 (FEN1‐wt) and mutant type FEN1 (FEN1‐mut) were constructed and co‐transfected into 22Rv1 cells. Luciferase reporter assays showed that ELK1 overexpression increased the transcription activity of the FEN1 promoter but had no effect on pGL3‐FEN1 (mut) luciferase activity (Figure 7C). These results suggest that ELK1 regulates FEN1 transcription.

Taken together, these results indicate that AR knockdown may enhance the chemosensitivity of prostate cancer cells by downregulating FEN1 through the ERK/ELK1 signalling pathway.

## 4 DISCUSSION

Prostate cancer is a heterogeneous disease throughout its natural history, and its incidence is influenced by factors such as age, race and heredity. Prognostic effects and biological characteristics vary greatly among individuals.^28^ In recent years, radiogenomics has achieved satisfactory results in characterising prostate cancer heterogeneity and improving the accuracy of diagnosis and prognosis.^29^ By targeting the androgen signalling axis, ADT effectively treats advanced and metastatic prostate cancer. However, prostate tumours can evade ADT in many ways, resulting in CRPC. Nonetheless, AR remains a key therapeutic target for CRPC. New non‐steroidal AR blockers, such as enzalutamide and apalutamide, have shown encouraging therapeutic effects in the treatment of CRPC.^30^, ^31^ Currently, chemotherapy is the mainstay adjuvant treatment for most patients with CRPC. As a taxane antitumor drug, DTX strengthens tubulin polymerisation and inhibits microtubule depolymerisation, thus hindering tumour cell mitosis, which ultimately leads to cell cycle arrest and apoptosis.^32^ Additionally, as a second‐generation taxane, cabazitaxel remains clinically active in some patients with docetaxel‐resistant prostate cancer. Besides, higher Gleason score may predict better prognosis in CRPC patients treated with cabazitaxel.^33^ Unfortunately, prostate cancer death occurs due to treatment failure. Therefore, it is imperative to identify novel targets that can be used in conjunction with standard therapy to improve patient outcomes.

FEN1 is a structure‐specific endonuclease that participates in DNA repair and contributes to tumour progression, metastasis and drug resistance in a variety of tumour types. Although previous studies have indicated that FEN1 promotes cell proliferation in prostate cancer,^22^ the role of FEN1 in the prostate cancer progression, metastasis and drug resistance remains unclear. Through bioinformatics analyses, we confirmed that FEN1 is upregulated in prostate cancer and is associated with a poor prognosis. Notably, a positive correlation between AR and FEN1 expression has likewise been observed in prostate cancer. We further found that testosterone promoted AR and FEN1 expression in prostate cancer cells, while DTX treatment yielded the opposite results. Lu et al. has confirmed that DTX inhibits AR activity in CRPC cell lines by inducing nuclear accumulation of FOXO1 (a known AR inhibitory nuclear factor).^34^ Zhu further suggested that DTX inhibits androgen‐dependent AR nuclear translocation by targeting the association of AR with tubulin.^35^ Therefore, AR inhibition is a potential mechanism for DTX within prostate cancer treatment.

In the present study, both in vivo and in vitro experiments showed that FEN1 inhibited DTX‐induced apoptosis and reversed G1/S phase arrest caused by DTX in prostate cancer, which eventually promoted tumour growth; knocking down FEN1 yielded the opposite results. These results demonstrate that blocking FEN1 enhances the sensitivity of prostate cancer cells to DTX. Similar results have been confirmed for other types of malignant tumours. For example, FEN1 blockade re‐sensitises platinum‐resistant ovarian cancer cells to cisplatin.^17^ In breast cancer, microRNA‐140 enhanced the chemotherapeutic response by targeting FEN1.^21^ Additionally, FEN1 inhibitors sensitise lung cancer cells to cisplatin and efficiently suppress cancer progression.^36^ Since FEN1 plays important roles in long‐patch BER and Okazaki fragment maturation,^15^ it could substantially attenuate the anti‐tumour effects of DNA damaging agents, such as cisplatin and DTX. In addition, recent studies have also shown that FEN1 knockdown induces apoptosis through the activation of autophagy, which enhances the anticancer effects of epirubicin in osteosarcoma.^37^ Inhibition of FEN1 could increase arsenic trioxide‐induced ROS accumulation and cell death in triple‐negative breast cancer.^20^ Although DTX mainly inhibits cell mitosis and induces cell cycle arrest, our results showed that FEN1 knockdown enhances the effect of DTX by inducing G1/S phase arrest. In non‐small cell lung cancer, cell cycle arrest at the G1/S or G2/M phase was induced after FEN1 knockdown.^19^ Dysregulation of FEN1 activity could result in the destruction of genetic information as well as disorders within the programmed cell cycle.^38^ In the normal cell cycle, FEN1 expression increases at the G1 and S phases and decreases sharply during the G2/M phase.^38^ Phosphorylation‐deficient FEN1 mutation impairs oxidative DNA damage repair and causes cell cycle arrest.^39^ Therefore, we speculate that FEN1 knockdown leads to unrepaired DNA damage induced by DTX in prostate cancer cells, which results in cell cycle arrest at the S phase and increased cell apoptosis.

As a key therapeutic target, there is no doubt that AR plays an important role in prostate cancer development, metastasis and drug resistance.^27^ In addition, many studies have shown that AR signalling can inhibit the sensitivity of prostate cancer to DTX treatment.^40^, ^41^ For example, the CHAARTED and STAMPEDE clinical studies have shown that, for patients with metastatic hormone‐sensitive prostate cancer, ADT combined with DTX can statistically significantly reduce the patient's risk of death and prolong imaging progression‐free survival time.^42^, ^43^ Consistent with the above results, our results demonstrate that AR knockdown enhances DTX‐induced apoptosis and cell cycle arrest at the S phase in prostate cancer. Moreover, Western blot and IHC experiments confirmed that AR knockdown inhibits FEN1 and MAPK/ERK signalling pathway expression. In particular, FEN1 overexpression reversed apoptosis and cell cycle arrest in prostate cancer cells due to the synergistic effects of AR knockdown and DTX. These results reveal that AR knockdown improves DTX sensitivity in prostate cancer cells by downregulating FEN1, while the MAPK/ERK signalling pathway may play a particularly important role during this process. The MAPK cascade is a key signalling pathway that regulates proliferation, survival and differentiation of normal cells. This pathway is frequently abnormally activated in malignant tumours.^44^ ERK is a downstream component of the evolutionarily conserved signal module activated by Raf serine/threonine kinase. RAF activates MAPK/ERK kinase, which in turn activates ERK1/2.^45^ In prostate cancer, the MAPK/ERK signalling pathway is confirmed to be involved in tumorigenesis, progression, metastasis and drug resistance.^45^, ^46^, ^47^ Moreover, the MAPK/ERK signalling pathway has been proposed as the central pathway for AR‐mediated non‐genomic regulation of prostate cancer cell proliferation.^25^ In this study, we found that the MEK inhibitor U0126 inhibits FEN1 protein levels in prostate cancer. Furthermore, the luciferase reporter assay results demonstrated that ELK1 binds for the FEN1 promoter and can activate the transcription of FEN1. To our knowledge, this is the first study to explore the association between the MAPK/ERK signalling pathway and FEN1. Wang et al. found that letrozole inhibits FEN1 expression in an ERK/ELK‐1‐dependent manner in breast cancer cells.^48^ However, Wang's research did not explore whether activated ELK‐1 binds to the FEN1 promoter. Therefore, we carried out supplementary experiments on this research question and confirmed this regulatory relationship. AR blockade not only synergizes with DTX in prostate cancer, clinical studies have also shown that abiraterone combined with olaparib therapy has achieved satisfactory results in mCRPC patients.^49^, ^50^ AR blocking treatment leads to decreased cellular DNA repair and increased levels of dsDNA breaks, which presents an opportunity for combining PARP inhibitors.^51^ As a structure‐specific endonuclease involved in DNA repair, inhibition of FEN1 may also work synergistically with PARP inhibitors, which deserves further study. Lipid metabolism has been proved to be involved in the resistance of prostate cancer to ADT and chemotherapy.^52^, ^53^ Moreover, AR is intimately involved in many lipogenesis processes.^54^ Therefore, key regulators of lipid metabolism may serve as new biomarkers or new therapeutic targets.^55^

Although we have studied the role of AR and FEN1 in prostate cancer extensively and confirmed the non‐genomic regulatory mechanism of AR on FEN1, there are still several limitations to this work which should be emphasised. First, we still do not know whether AR is involved in the regulation of FEN1 phosphorylation or ubiquitin in prostate cancer. Second, whether phosphorylation and ubiquitination of FEN1 influences survival and cell cycle changes in prostate cancer cells remains unclear. Third, our current study lacks experimental research data on whether AR directly regulates FEN1.

**FIGURE 8:**
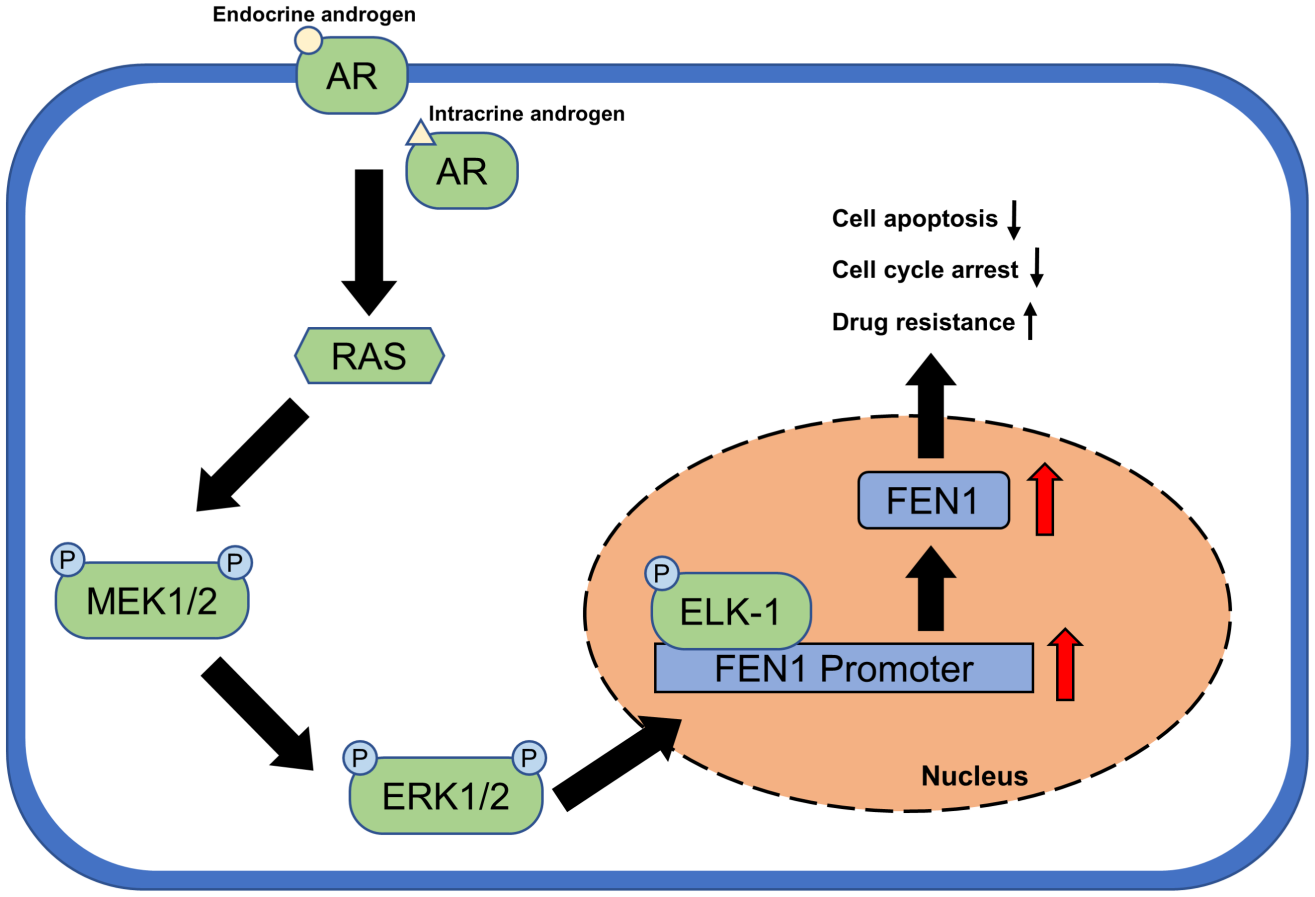
Mechanism of AR‐mediated regulation of FEN1 expression in prostate cancer. AR, androgen receptor; RAS, rat sarcoma oncogene; ERK, extracellular signal‐related kinase; MEK, mitogen‐activated protein/ERK kinase; ELK, ETS transcription factor 1; FEN1, flap structure‐specific endonuclease 1.

In conclusion, AR knockdown results in the inactivation of the ERK/ELK‐1 signalling pathway that, in turn, leads to the downregulation of FEN1 and improves the DTX sensitivity of prostate cancer cells (Figure. 8). Our research confirms a new mechanism of AR blocking in the treatment of prostate cancer and offers FEN1 as a promising anticancer therapeutic target for prostate cancer treatment in order to overcome DTX resistance.

## AUTHOR CONTRIBUTIONS

**Weijie Xie:** Conceptualization (lead); formal analysis (lead); methodology (lead); validation (lead); writing – original draft (lead); writing – review and editing (equal). **ShuLin Li:** Formal analysis (equal); methodology (equal); validation (equal). **Huan Guo:** Formal analysis (equal); methodology (equal); validation (equal). **Jiawei Zhang:** Methodology (equal); validation (equal). **Menjiang Tu:** Methodology (equal); validation (equal). **Rui Wang:** Funding acquisition (equal); methodology (equal); validation (equal). **Bingling Lin:** Formal analysis (equal); funding acquisition (equal); writing – review and editing (equal). **Yuqi Wu:** Conceptualization (equal); formal analysis (equal); writing – review and editing (lead). **Xiangwei Wang:** Funding acquisition (lead); supervision (lead); writing – review and editing (equal).

## FUNDING INFORMATION

The study was sponsored by the Science and Technology Innovation Commission Foundation of Shenzhen (Grant No. JCYJ20190808141013454 and JCYJ20180305124827261), National key R&D program (Grant No. 2020YFA0908800), Shenzhen Key Laboratory Foundation (Grant No. ZDSYS20200811143757022), Nanshan District Health Science and Technology Project (Grant No. NS2021159) and the National Natural Science Foundation of China (Grant No. 82102119). The funding body played no role in the design of the study; collection, analysis, and interpretation of data; and writing of the manuscript.

## CONFLICT OF INTEREST STATEMENT

The author reports no conflicts of interest in this work.

## Supporting information


Data S1.

## Data Availability

Tha datasets used for bioinformatics analyses are available in the TCGA (https://portal.gdc.cancer.gov/) and GEO (https://www.ncbi.nlm.nih.gov/geo/). Other data and materials used to support the findings of this study are available upon request from the corresponding author.
