# Supplementary material for: Androgen receptor knockdown enhances prostate cancer chemosensitivity by down‐regulating FEN1 through the ERK/ELK1 signalling pathway
Source: Cancer Med. 2023 Jun 16;12(14):15317–36. doi: 10.1002/cam4.6188 (PMC10417077; doi:10.1002/cam4.6188)
Supplement: Supplementary file 1 — Data S1. [file CAM4-12-15317-s001.docx]

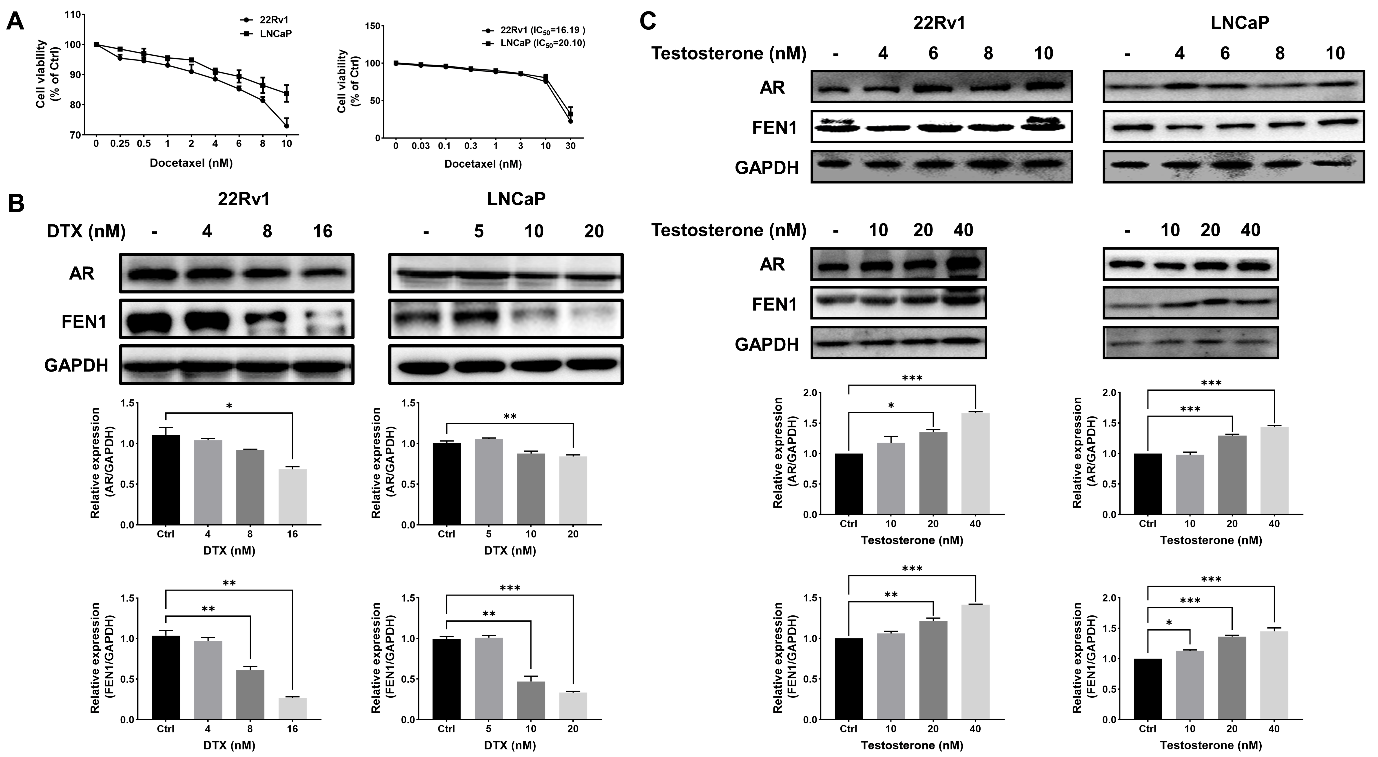


**Supplementary Figure 1.** Effects of testosterone and docetaxel (DTX) on cell viability and androgen receptor (AR) and flap endonuclease 1 (FEN1) expression in 22Rv1 and LNCaP cells. **(A)** IC50 curve shows the effect of DTX on cell viability in 22Rv1 and LNCaP cells. **(B)** Western blot results showing that DTX decreased AR and FEN1 expression in 22Rv1 and LNCaP cells. **(C)** Western blot results showing testosterone increased AR and FEN1 expression in 22Rv1 and LNCaP cells. Data are presented as the mean ± SD values, and the error bars represent data from triplicate biological experiments. **P*<0.05, ***P*<0.01, ****P*<0.001

**
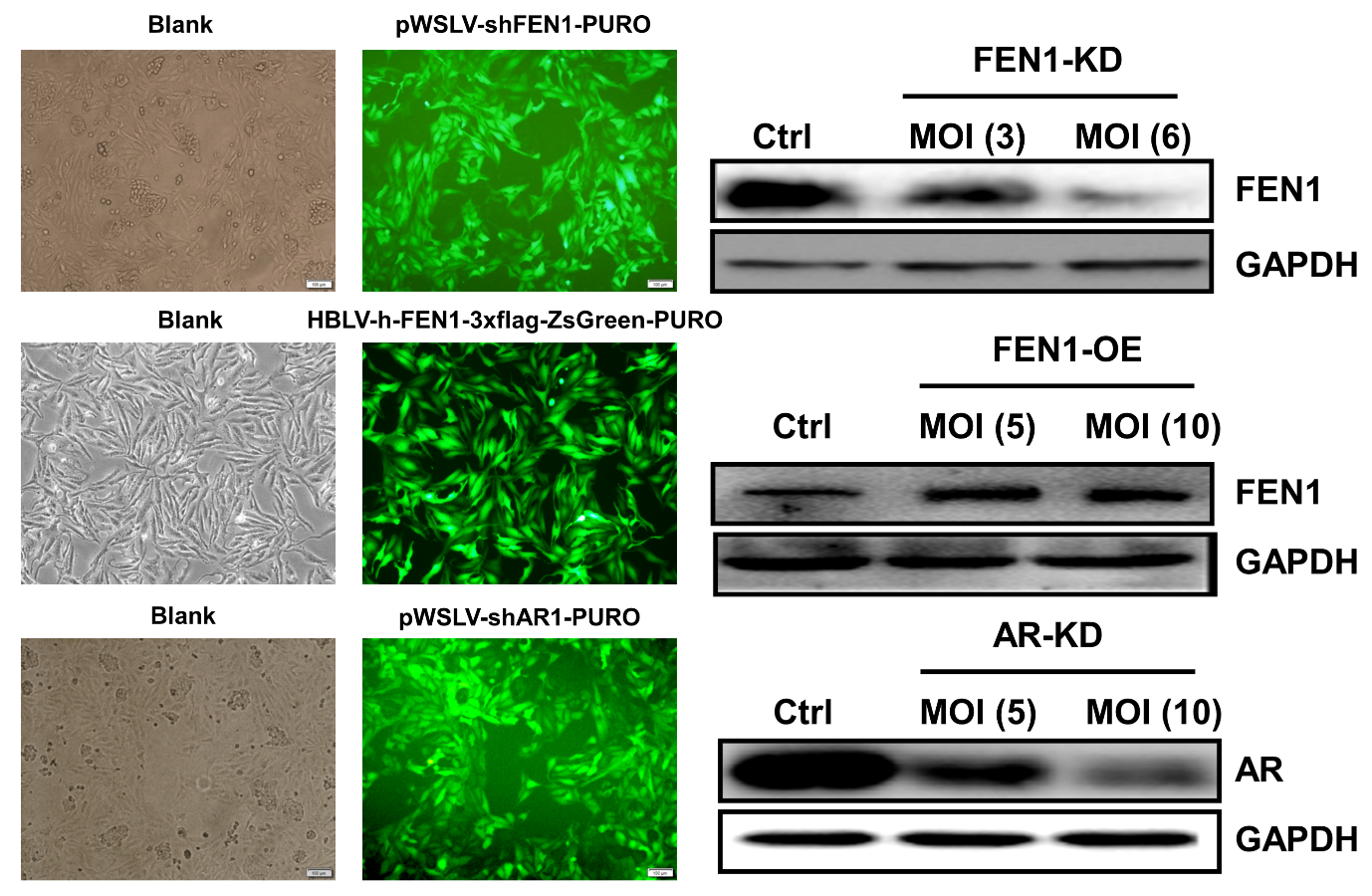
**

**Supplementary Figure 2.** Construction of 22Rv1 cells with stable flap structure-specific endonuclease 1 (FEN1) overexpression and knockdown or androgen receptor (AR) knockdown. Western blot was used to verify the construction of stable cell lines.


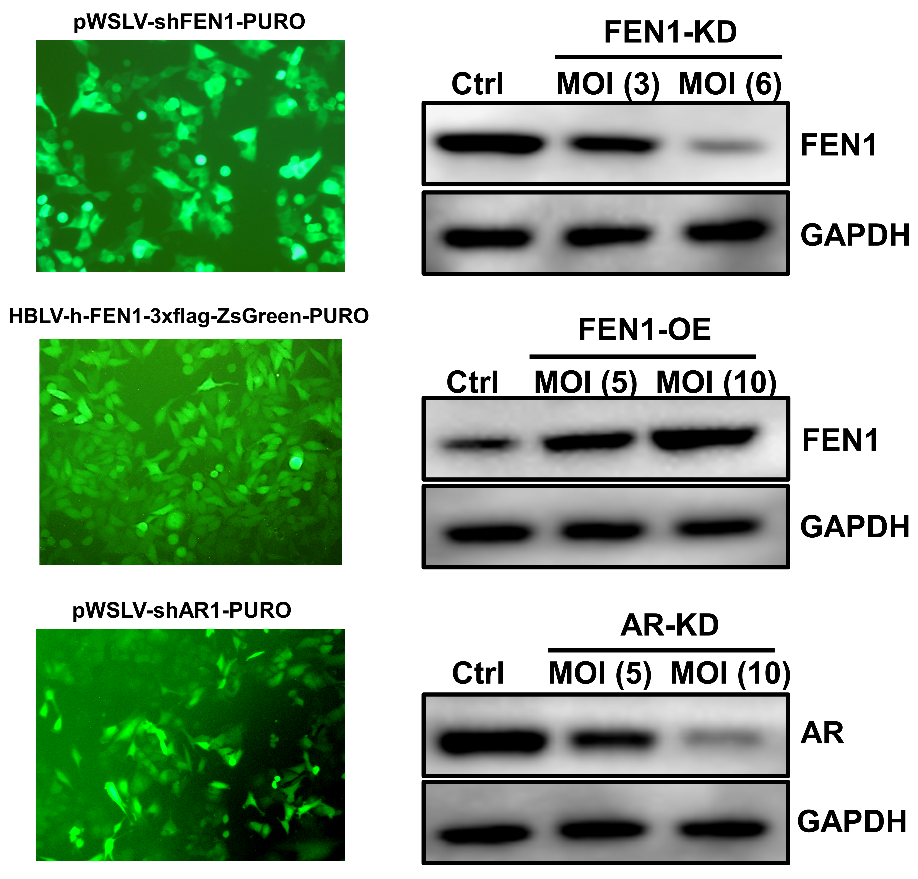


**Supplementary Figure 3.** Construction of LNCaP cells with stable flap structure-specific endonuclease 1 (FEN1) overexpression and knockdown or androgen receptor (AR) knockdown. Western blot was used to verify the construction of stable cell lines.


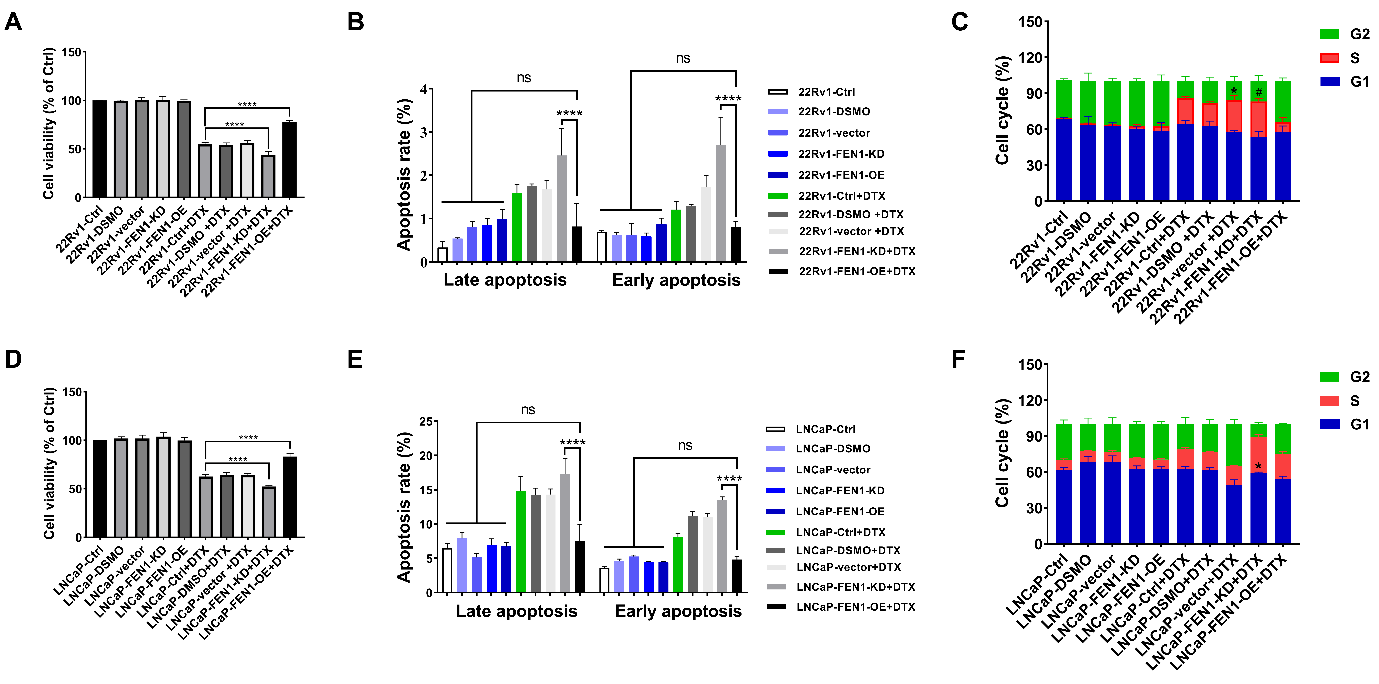


**Supplementary Figure 4.** **Effects of FEN1 overexpression or konckdown on cell viability, cell apoptosis, and cell cycle in 22Rv1 and LNCaP cells**. (A) Effects of FEN1 expression on cell viability in 22Rv1 cells treated with docetaxel (DTX). *****P*<0.0001. (B) Effects of FEN1 expression on apoptosis in 22Rv1 cells treated with DTX. *****P*<0.0001. (C) Effects of FEN1 expression on cell cycle in 22Rv1 cells treated with DTX. **P*<0.05, 22Rv1-vector+DTX vs. 22Rv1-FEN1-OE+DTX; ^#^*P*<0.05, 22Rv1-FEN1-KD+DTX vs. 22Rv1-FEN1-OE+DTX. (D) Effect sof FEN1 expression on cell viability in LNCaP cells treated with DTX. *****P*<0.0001. (E) Effects of FEN1 expression on apoptosis in LNCaP cells treated with DTX. *****P*<0.0001. (F) Effects of FEN1 expression on cell cycle in LNCaP cells treated with DTX. **P*<0.05, LNCaP-FEN1-KD+DTX vs. LNCaP-Ctrl+DTX, LNCaP-DMSO+DTX, and LNCaP-vector+DTX groups. **P*<0.05, ^#^*P*<0.05, ***P*<0.01, ****P*<0.001, *****P*<0.0001, ns, no significance. 22Rv1 cells were devided into the following groups: 22Rv1-Ctrl, 22Rv1-Ctrl+DTX, 22Rv1-DSMO, 22Rv1-DSMO+DTX, 22Rv1-vector, 22Rv1-vector+DTX, 22Rv1-FEN1-KD group, 22Rv1-FEN1-KD+DTX, 22Rv1-FEN1-OE, and 22Rv1-FEN1-OE+DTX groups. LNCaP cells were similarly grouped. DMSO, Dimethyl sulfoxide


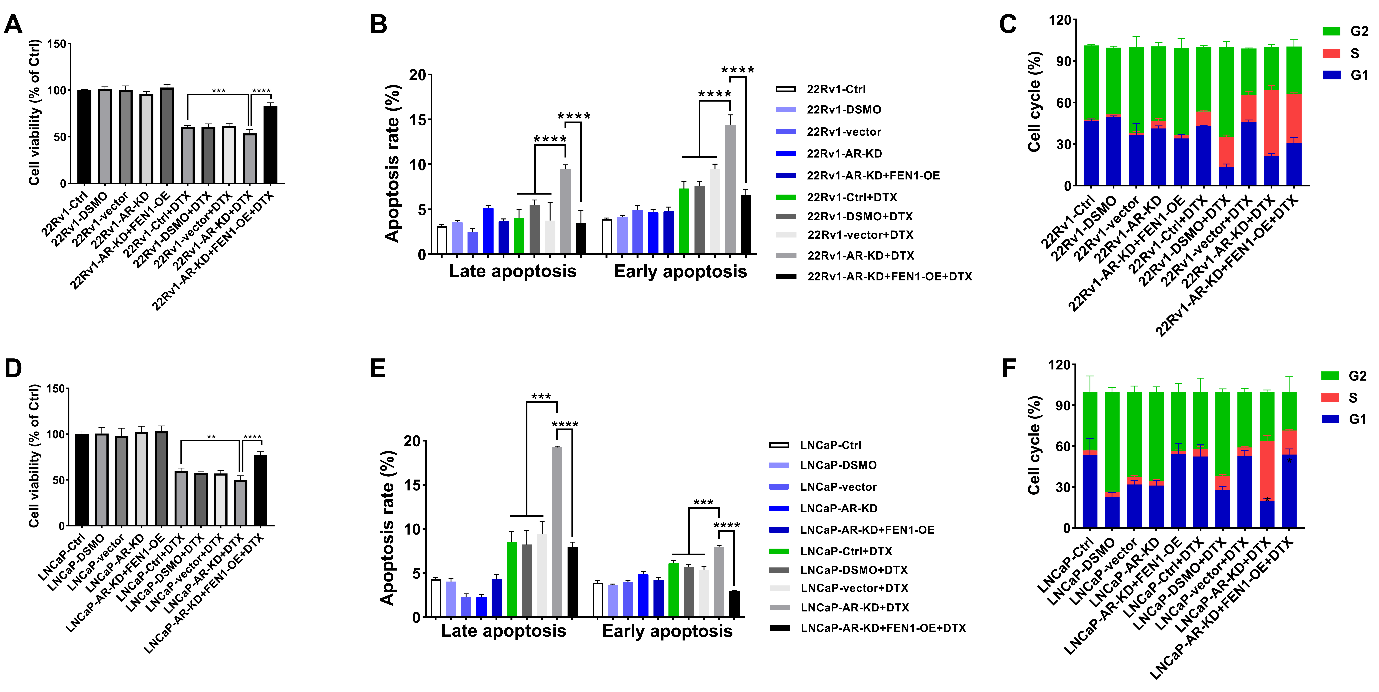


**Supplementary Figure 5. Effects of AR konckdown and FEN1 overexpression on cell viability, cell apoptosis, and cell cycle in 22Rv1 and LNCaP cells treated with DTX.** (A) Effects of AR konckdown and FEN1 overexpression on cell viability in 22Rv1 cells treated with DTX. ****P*<0.001, *****P*<0.0001. (B) Effects of AR silencing and FEN1 overexpression on apoptosis in 22Rv1 cells treated with DTX. *****P*<0.0001. (C) Effects of AR konckdown and FEN1 overexpression on cell cycle in 22Rv1 cells treated with DTX. (D) Effects of AR konckdown and FEN1 overexpression on cell viability in LNCaP cells treated with DTX. ***P*<0.01, *****P*<0.0001. (E) Effects of AR konckdown and FEN1 overexpression on apoptosis in LNCaP cells treated with DTX. ****P*<0.001, *****P*<0.0001. (F) Effects of AR konckdown and FEN1 overexpression on LNCaP cells cycle treated with DTX. **P*<0.05, ***P*<0.01, ****P*<0.001, *****P*<0.0001, ns, no significance. 22Rv1 cells were devided into the following groups: 22Rv1-Ctrl, 22Rv1-DSMO, 22Rv1-AR-KD, 22Rv1-vector, 22Rv1-AR-KD+FEN1-OE group, 22Rv1-Ctrl+DTX group, 22Rv1-DSMO+DTX, 22Rv1-vector+DTX, 22Rv1-AR-KD+DTX, and 22Rv1-AR-KD+FEN1-OE+DTX groups. LNCaP cells were similarly grouped.

**Sequence details of FEN1-wt promoters:**

**基因合成报告单**

| 样品编号 | Abs260 | Abs280 | Abs230 | 260/230 | 260/280 | 样品浓度 |
| --- | --- | --- | --- | --- | --- | --- |
| E8650-4 | 2.883 | 1.562 | 1.449 | 1.99 | 1.85 | 144.13 |

| 基因名称:BK640 PGL3-Basic-FEN1-p-WT  捷瑞编号:PA5174Gn  克隆编号:E8650-4  接单日期:2020/4/23  交货日期:2020/5/12  基因长度:1012 bp  克隆载体:pGL3-basic  克隆位点:NheI/XhoI  克隆载体抗性:Amp  测序反应:2  交付材料:1 x 5ug质粒，冰箱-20℃保存 |
| --- |

**注意事项**

1．DNA质粒是从包含甲基化酶的大肠杆菌提取分离而得，因此受甲基化作用限制的内切酶被锁住；

2．可根据产品的综合序列测序图，显示合成产品的正确序列

## **质量控制：**


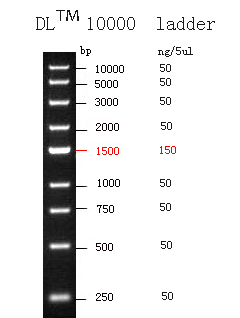


Line2:digested with NheI/XhoI

Line1:ladder


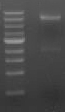


基因名称:BK640 PGL3-Basic-FEN1-p-WT

克隆编号:E8650-4

## 结构图：


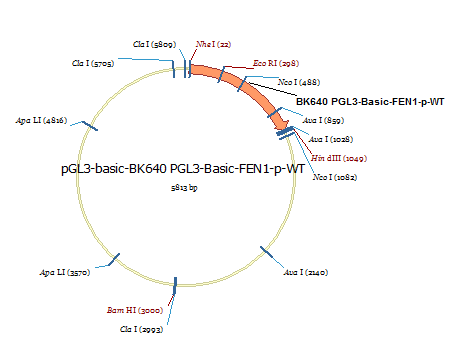


## **全序列：(正向)**

| 1  51  101  151  201  251  301  351  401  451  501  551  601  651  701  751  801  851  901  951  1001  1051  1101  1151  1201  1251  1301  1351  1401  1451  1501  1551  1601  1651  1701  1751  1801  1851  1901  1951  2001  2051  2101  2151  2201  2251  2301  2351  2401  2451  2501  2551  2601  2651  2701  2751  2801  2851  2901  2951  3001  3051  3101  3151  3201  3251  3301  3351  3401  3451  3501  3551  3601  3651  3701  3751  3801  3851  3901  3951  4001  4051  4101  4151  4201  4251  4301  4351  4401  4451  4501  4551  4601  4651  4701  4751  4801  4851  4901  4951  5001  5051  5101  5151  5201  5251  5301  5351  5401  5451  5501  5551  5601  5651  5701  5751  5801  5851  5901 | GGTACCGAGC TCTTACGCGT GCTAGCTGTA ATATCTATTT CACAGAACTG  AAAAATAAGA AAGATGATGA ATCAAAGCAT CTAGTGCCTA GCAGGGAGTA  TTTTGCTCAA CAGGTATTTG CTTCCTTCCT AAGGCTGTAG GGAAGATGAT  GAGATAATGT CTTTTATGAA AGAGGGCTGT AAACGTAAAG ATCTGTACAA  ATGTTAACTT CATTGTCACC GGTCAGCCAA TGCTTCTAAA ATCCAGAACA  TAACAACTCT AGAGAAGTAA ACTGCCCCCA TTGTTCTGAG ACACTGGAAT  TCAATTCAGT AAACAATCAC GGCCCCCTTC CCCCAAAATG ATAAAGACAA  TCACTGCCAT TTATTGAGCT TCCAATTACG GGCCCTCTGT TTGGCACTGA  GAATACAAAG ATGAATAGAC ATCATCCCAG AGCTAGATGC GCGTCAGACG  GTGGTCACTA GGAGGCGTGG CCGAAAACAA AGAAGTCCAT GGAACGTGGC  CAGAGATCTG TACAGAGGCT GTGGGCGCTC CTAGGAAAGT CTGGCCAAGT  GCCTGAGAGT TGGAAGTGCT TCACCAATAA ACATTTGCCC AGGGCATTGT  AGGATGGGCA CGGGTTCGGC AGAAGAACTT TCCAAATAAA GATAACACAC  CACCGATAAC AGAGATATAC AAACTGGAAG GTATTCAAAA TTCGCCCCAC  GCCTCTCGCC CTTAGAAATC GCGAGCTGAG AAACCTAAGG AGTTCATGGC  AAGGGGCTTC CCCCTTCCCC ACCCTTCAGC CCAAGCCGGA GGTTCCAGGA  GCGTCTAGCC CTCTGGATCT CCGGCGTCTG AGGAGATAAG CGCGGTGTGG  GTCAGACCCC GAGGGGTCCT CGCATCTCCG TCTGGAACTC CCCTCAACGC  TCTCACCATT TTGCCCCGCG AAGGCTAATC CGCCGCTCCG CCACCGGAAG  AACACGTCGG CAGGAGCAGG CGCCTAGCAC AACCGGAAAA GGAAGTGCCT  CCGGCGCAAG TGGCATTGAG GGACTTCTCG AGATCTGCGA TCTAAGTAAG  CTTGGCATTC CGGTACTGTT GGTAAAGCCA CCATGGAAGA CGCCAAAAAC  ATAAAGAAAG GCCCGGCGCC ATTCTATCCG CTGGAAGATG GAACCGCTGG  AGAGCAACTG CATAAGGCTA TGAAGAGATA CGCCCTGGTT CCTGGAACAA  TTGCTTTTAC AGATGCACAT ATCGAGGTGG ACATCACTTA CGCTGAGTAC  TTCGAAATGT CCGTTCGGTT GGCAGAAGCT ATGAAACGAT ATGGGCTGAA  TACAAATCAC AGAATCGTCG TATGCAGTGA AAACTCTCTT CAATTCTTTA  TGCCGGTGTT GGGCGCGTTA TTTATCGGAG TTGCAGTTGC GCCCGCGAAC  GACATTTATA ATGAACGTGA ATTGCTCAAC AGTATGGGCA TTTCGCAGCC  TACCGTGGTG TTCGTTTCCA AAAAGGGGTT GCAAAAAATT TTGAACGTGC  AAAAAAAGCT CCCAATCATC CAAAAAATTA TTATCATGGA TTCTAAAACG  GATTACCAGG GATTTCAGTC GATGTACACG TTCGTCACAT CTCATCTACC  TCCCGGTTTT AATGAATACG ATTTTGTGCC AGAGTCCTTC GATAGGGACA  AGACAATTGC ACTGATCATG AACTCCTCTG GATCTACTGG TCTGCCTAAA  GGTGTCGCTC TGCCTCATAG AACTGCCTGC GTGAGATTCT CGCATGCCAG  AGATCCTATT TTTGGCAATC AAATCATTCC GGATACTGCG ATTTTAAGTG  TTGTTCCATT CCATCACGGT TTTGGAATGT TTACTACACT CGGATATTTG  ATATGTGGAT TTCGAGTCGT CTTAATGTAT AGATTTGAAG AAGAGCTGTT  TCTGAGGAGC CTTCAGGATT ACAAGATTCA AAGTGCGCTG CTGGTGCCAA  CCCTATTCTC CTTCTTCGCC AAAAGCACTC TGATTGACAA ATACGATTTA  TCTAATTTAC ACGAAATTGC TTCTGGTGGC GCTCCCCTCT CTAAGGAAGT  CGGGGAAGCG GTTGCCAAGA GGTTCCATCT GCCAGGTATC AGGCAAGGAT  ATGGGCTCAC TGAGACTACA TCAGCTATTC TGATTACACC CGAGGGGGAT  GATAAACCGG GCGCGGTCGG TAAAGTTGTT CCATTTTTTG AAGCGAAGGT  TGTGGATCTG GATACCGGGA AAACGCTGGG CGTTAATCAA AGAGGCGAAC  TGTGTGTGAG AGGTCCTATG ATTATGTCCG GTTATGTAAA CAATCCGGAA  GCGACCAACG CCTTGATTGA CAAGGATGGA TGGCTACATT CTGGAGACAT  AGCTTACTGG GACGAAGACG AACACTTCTT CATCGTTGAC CGCCTGAAGT  CTCTGATTAA GTACAAAGGC TATCAGGTGG CTCCCGCTGA ATTGGAATCC  ATCTTGCTCC AACACCCCAA CATCTTCGAC GCAGGTGTCG CAGGTCTTCC  CGACGATGAC GCCGGTGAAC TTCCCGCCGC CGTTGTTGTT TTGGAGCACG  GAAAGACGAT GACGGAAAAA GAGATCGTGG ATTACGTCGC CAGTCAAGTA  ACAACCGCGA AAAAGTTGCG CGGAGGAGTT GTGTTTGTGG ACGAAGTACC  GAAAGGTCTT ACCGGAAAAC TCGACGCAAG AAAAATCAGA GAGATCCTCA  TAAAGGCCAA GAAGGGCGGA AAGATCGCCG TGTAATTCTA GAGTCGGGGC  GGCCGGCCGC TTCGAGCAGA CATGATAAGA TACATTGATG AGTTTGGACA  AACCACAACT AGAATGCAGT GAAAAAAATG CTTTATTTGT GAAATTTGTG  ATGCTATTGC TTTATTTGTA ACCATTATAA GCTGCAATAA ACAAGTTAAC  AACAACAATT GCATTCATTT TATGTTTCAG GTTCAGGGGG AGGTGTGGGA  GGTTTTTTAA AGCAAGTAAA ACCTCTACAA ATGTGGTAAA ATCGATAAGG  ATCCGTCGAC CGATGCCCTT GAGAGCCTTC AACCCAGTCA GCTCCTTCCG  GTGGGCGCGG GGCATGACTA TCGTCGCCGC ACTTATGACT GTCTTCTTTA  TCATGCAACT CGTAGGACAG GTGCCGGCAG CGCTCTTCCG CTTCCTCGCT  CACTGACTCG CTGCGCTCGG TCGTTCGGCT GCGGCGAGCG GTATCAGCTC  ACTCAAAGGC GGTAATACGG TTATCCACAG AATCAGGGGA TAACGCAGGA  AAGAACATGT GAGCAAAAGG CCAGCAAAAG GCCAGGAACC GTAAAAAGGC  CGCGTTGCTG GCGTTTTTCC ATAGGCTCCG CCCCCCTGAC GAGCATCACA  AAAATCGACG CTCAAGTCAG AGGTGGCGAA ACCCGACAGG ACTATAAAGA  TACCAGGCGT TTCCCCCTGG AAGCTCCCTC GTGCGCTCTC CTGTTCCGAC  CCTGCCGCTT ACCGGATACC TGTCCGCCTT TCTCCCTTCG GGAAGCGTGG  CGCTTTCTCA TAGCTCACGC TGTAGGTATC TCAGTTCGGT GTAGGTCGTT  CGCTCCAAGC TGGGCTGTGT GCACGAACCC CCCGTTCAGC CCGACCGCTG  CGCCTTATCC GGTAACTATC GTCTTGAGTC CAACCCGGTA AGACACGACT  TATCGCCACT GGCAGCAGCC ACTGGTAACA GGATTAGCAG AGCGAGGTAT  GTAGGCGGTG CTACAGAGTT CTTGAAGTGG TGGCCTAACT ACGGCTACAC  TAGAAGAACA GTATTTGGTA TCTGCGCTCT GCTGAAGCCA GTTACCTTCG  GAAAAAGAGT TGGTAGCTCT TGATCCGGCA AACAAACCAC CGCTGGTAGC  GGTGGTTTTT TTGTTTGCAA GCAGCAGATT ACGCGCAGAA AAAAAGGATC  TCAAGAAGAT CCTTTGATCT TTTCTACGGG GTCTGACGCT CAGTGGAACG  AAAACTCACG TTAAGGGATT TTGGTCATGA GATTATCAAA AAGGATCTTC  ACCTAGATCC TTTTAAATTA AAAATGAAGT TTTAAATCAA TCTAAAGTAT  ATATGAGTAA ACTTGGTCTG ACAGTTACCA ATGCTTAATC AGTGAGGCAC  CTATCTCAGC GATCTGTCTA TTTCGTTCAT CCATAGTTGC CTGACTCCCC  GTCGTGTAGA TAACTACGAT ACGGGAGGGC TTACCATCTG GCCCCAGTGC  TGCAATGATA CCGCGAGACC CACGCTCACC GGCTCCAGAT TTATCAGCAA  TAAACCAGCC AGCCGGAAGG GCCGAGCGCA GAAGTGGTCC TGCAACTTTA  TCCGCCTCCA TCCAGTCTAT TAATTGTTGC CGGGAAGCTA GAGTAAGTAG  TTCGCCAGTT AATAGTTTGC GCAACGTTGT TGCCATTGCT ACAGGCATCG  TGGTGTCACG CTCGTCGTTT GGTATGGCTT CATTCAGCTC CGGTTCCCAA  CGATCAAGGC GAGTTACATG ATCCCCCATG TTGTGCAAAA AAGCGGTTAG  CTCCTTCGGT CCTCCGATCG TTGTCAGAAG TAAGTTGGCC GCAGTGTTAT  CACTCATGGT TATGGCAGCA CTGCATAATT CTCTTACTGT CATGCCATCC  GTAAGATGCT TTTCTGTGAC TGGTGAGTAC TCAACCAAGT CATTCTGAGA  ATAGTGTATG CGGCGACCGA GTTGCTCTTG CCCGGCGTCA ATACGGGATA  ATACCGCGCC ACATAGCAGA ACTTTAAAAG TGCTCATCAT TGGAAAACGT  TCTTCGGGGC GAAAACTCTC AAGGATCTTA CCGCTGTTGA GATCCAGTTC  GATGTAACCC ACTCGTGCAC CCAACTGATC TTCAGCATCT TTTACTTTCA  CCAGCGTTTC TGGGTGAGCA AAAACAGGAA GGCAAAATGC CGCAAAAAAG  GGAATAAGGG CGACACGGAA ATGTTGAATA CTCATACTCT TCCTTTTTCA  ATATTATTGA AGCATTTATC AGGGTTATTG TCTCATGAGC GGATACATAT  TTGAATGTAT TTAGAAAAAT AAACAAATAG GGGTTCCGCG CACATTTCCC  CGAAAAGTGC CACCTGACGC GCCCTGTAGC GGCGCATTAA GCGCGGCGGG  TGTGGTGGTT ACGCGCAGCG TGACCGCTAC ACTTGCCAGC GCCCTAGCGC  CCGCTCCTTT CGCTTTCTTC CCTTCCTTTC TCGCCACGTT CGCCGGCTTT  CCCCGTCAAG CTCTAAATCG GGGGCTCCCT TTAGGGTTCC GATTTAGTGC  TTTACGGCAC CTCGACCCCA AAAAACTTGA TTAGGGTGAT GGTTCACGTA  GTGGGCCATC GCCCTGATAG ACGGTTTTTC GCCCTTTGAC GTTGGAGTCC  ACGTTCTTTA ATAGTGGACT CTTGTTCCAA ACTGGAACAA CACTCAACCC  TATCTCGGTC TATTCTTTTG ATTTATAAGG GATTTTGCCG ATTTCGGCCT  ATTGGTTAAA AAATGAGCTG ATTTAACAAA AATTTAACGC GAATTTTAAC  AAAATATTAA CGCTTACAAT TTGCCATTCG CCATTCAGGC TGCGCAACTG  TTGGGAAGGG CGATCGGTGC GGGCCTCTTC GCTATTACGC CAGCCCAAGC  TACCATGATA AGTAAGTAAT ATTAAGGTAC GGGAGGTACT TGGAGCGGCC  GCAATAAAAT ATCTTTATTT TCATTACATC TGTGTGTTGG TTTTTTGTGT  GAATCGATAG TACTAACATA CGCTCTCCAT CAAAACAAAA CGAAACAAAA  CAAACTAGCA AAATAGGCTG TCCCCAGTGC AAGTGCAGGT GCCAGAACAT  TTCTCTATCG ATA |
| --- | --- |

**Sequence details of FEN1-mut promoters:**

**基因合成报告单**

| 样品编号 | Abs260 | Abs280 | Abs230 | 260/230 | 260/280 | 样品浓度 |
| --- | --- | --- | --- | --- | --- | --- |
| E8249-1 | 7.12 | 3.976 | 3.538 | 2.01 | 1.79 | 356.01 |

| 基因名称:BK641 PGL3-Basic-FEN1-p-MU1  捷瑞编号:PA5175Gn  克隆编号:E8249-1  接单日期:2020/4/23  交货日期:2020/4/30  基因长度:1012 bp  克隆载体:pGL3-basic  克隆位点:NheI/XhoI  克隆载体抗性:Amp  测序反应:2  交付材料:1x 5ug质粒，冰箱-20℃保存 |
| --- |

**注意事项**

1．DNA质粒是从包含甲基化酶的大肠杆菌提取分离而得，因此受甲基化作用限制的内切酶被锁住；

2．可根据产品的综合序列测序图，显示合成产品的正确序列

## **质量控制：**


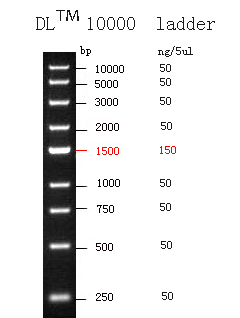


Line2:digested with NheI/XhoI

Line1:ladder


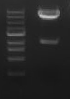


基因名称:BK641 PGL3-Basic-FEN1-p-MU1

克隆编号:E8249-1

## 结构图：


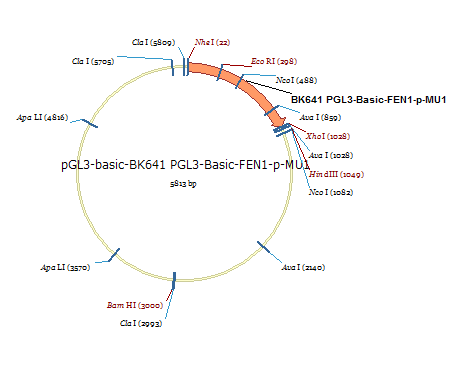


## **全序列：(正向)**

| 1  51  101  151  201  251  301  351  401  451  501  551  601  651  701  751  801  851  901  951  1001  1051  1101  1151  1201  1251  1301  1351  1401  1451  1501  1551  1601  1651  1701  1751  1801  1851  1901  1951  2001  2051  2101  2151  2201  2251  2301  2351  2401  2451  2501  2551  2601  2651  2701  2751  2801  2851  2901  2951  3001  3051  3101  3151  3201  3251  3301  3351  3401  3451  3501  3551  3601  3651  3701  3751  3801  3851  3901  3951  4001  4051  4101  4151  4201  4251  4301  4351  4401  4451  4501  4551  4601  4651  4701  4751  4801  4851  4901  4951  5001  5051  5101  5151  5201  5251  5301  5351  5401  5451  5501  5551  5601  5651  5701  5751  5801  5851  5901 | GGTACCGAGC TCTTACGCGT GCTAGCTGTA ATATCTATTT CACAGAACTG  AAAAATAAGA AAGATGATGA ATCAAAGCAT CTAGTGCCTA GCAGGGAGTA  TTTTGCTCAA CAGGTATTTG CTTCCTTCCT AAGGCTGTAG GGAAGATGAT  GAGATAATGT CTTTTATGAA AGAGGGCTGT AAACGTAAAG ATCTGTACAA  ATGTTAACTT CATTGTCACC GGTCAGCCAA TGCTTCTAAA ATCCAGAACA  TAACAACTCT AGAGAAGTAA ACTGCCCCCA TTGTTCTGAG ACACTGGAAT  TCAATTCAGT AAACAATCAC GGCCCCCTTC CCCCAAAATG ATAAAGACAA  TCACTGCCAT TTATTGAGCT TCCAATTACG GGCCCTCTGT TTGGCACTGA  GAATACAAAG ATGAATAGAC ATCATCCCAG AGCTAGATGC GCGTCAGACG  GTGGTCACTA GGAGGCGTGG CCGAAAACAA AGAAGTCCAT GGAACGTGGC  CAGAGATCTG TACAGAGGCT GTGGGCGCTC CTAGGAAAGT CTGGCCAAGT  GCCTGAGAGT TGGAAGTGCT TCACCAATAA ACATTTGCCC AGGGCATTGT  AGGATGGGCA CGGGTTCGGC AGAAGAACTT TCCAAATAAA GATAACACAC  CACCGATAAC AGAGATATAC AAACTGGAAG GTATTCAAAA TTCGCCCCAC  GCCTCTCGCC CTTAGAAATC GCGAGCTGAG AAACCTAAGG AGTTCATGGC  AAGGGGCTTC CCCCTTCCCC ACCCTTCAGC CCAAGCCGGA GGTTCCAGGA  GCGTCTAGCC CTCTGGATCT CCGGCGTCTG AGGAGATAAG CGCGGTGTGG  GTCAGACCCC GAGGGGTCCT CGCATCTCCG TCTGGAACTC CCCTCAACGC  TCTCACCATT TTGCCCCGCG AAGGCTAATC CGCCGCTCCG CCACCGGAAG  AACACGTCGG CAGGAGCAGG CGCCTAGCAC AACCGGAAAA GCAAGTGCCT  CCGGCGCAAG TGGCATTGAG GGACTTCTCG AGATCTGCGA TCTAAGTAAG  CTTGGCATTC CGGTACTGTT GGTAAAGCCA CCATGGAAGA CGCCAAAAAC  ATAAAGAAAG GCCCGGCGCC ATTCTATCCG CTGGAAGATG GAACCGCTGG  AGAGCAACTG CATAAGGCTA TGAAGAGATA CGCCCTGGTT CCTGGAACAA  TTGCTTTTAC AGATGCACAT ATCGAGGTGG ACATCACTTA CGCTGAGTAC  TTCGAAATGT CCGTTCGGTT GGCAGAAGCT ATGAAACGAT ATGGGCTGAA  TACAAATCAC AGAATCGTCG TATGCAGTGA AAACTCTCTT CAATTCTTTA  TGCCGGTGTT GGGCGCGTTA TTTATCGGAG TTGCAGTTGC GCCCGCGAAC  GACATTTATA ATGAACGTGA ATTGCTCAAC AGTATGGGCA TTTCGCAGCC  TACCGTGGTG TTCGTTTCCA AAAAGGGGTT GCAAAAAATT TTGAACGTGC  AAAAAAAGCT CCCAATCATC CAAAAAATTA TTATCATGGA TTCTAAAACG  GATTACCAGG GATTTCAGTC GATGTACACG TTCGTCACAT CTCATCTACC  TCCCGGTTTT AATGAATACG ATTTTGTGCC AGAGTCCTTC GATAGGGACA  AGACAATTGC ACTGATCATG AACTCCTCTG GATCTACTGG TCTGCCTAAA  GGTGTCGCTC TGCCTCATAG AACTGCCTGC GTGAGATTCT CGCATGCCAG  AGATCCTATT TTTGGCAATC AAATCATTCC GGATACTGCG ATTTTAAGTG  TTGTTCCATT CCATCACGGT TTTGGAATGT TTACTACACT CGGATATTTG  ATATGTGGAT TTCGAGTCGT CTTAATGTAT AGATTTGAAG AAGAGCTGTT  TCTGAGGAGC CTTCAGGATT ACAAGATTCA AAGTGCGCTG CTGGTGCCAA  CCCTATTCTC CTTCTTCGCC AAAAGCACTC TGATTGACAA ATACGATTTA  TCTAATTTAC ACGAAATTGC TTCTGGTGGC GCTCCCCTCT CTAAGGAAGT  CGGGGAAGCG GTTGCCAAGA GGTTCCATCT GCCAGGTATC AGGCAAGGAT  ATGGGCTCAC TGAGACTACA TCAGCTATTC TGATTACACC CGAGGGGGAT  GATAAACCGG GCGCGGTCGG TAAAGTTGTT CCATTTTTTG AAGCGAAGGT  TGTGGATCTG GATACCGGGA AAACGCTGGG CGTTAATCAA AGAGGCGAAC  TGTGTGTGAG AGGTCCTATG ATTATGTCCG GTTATGTAAA CAATCCGGAA  GCGACCAACG CCTTGATTGA CAAGGATGGA TGGCTACATT CTGGAGACAT  AGCTTACTGG GACGAAGACG AACACTTCTT CATCGTTGAC CGCCTGAAGT  CTCTGATTAA GTACAAAGGC TATCAGGTGG CTCCCGCTGA ATTGGAATCC  ATCTTGCTCC AACACCCCAA CATCTTCGAC GCAGGTGTCG CAGGTCTTCC  CGACGATGAC GCCGGTGAAC TTCCCGCCGC CGTTGTTGTT TTGGAGCACG  GAAAGACGAT GACGGAAAAA GAGATCGTGG ATTACGTCGC CAGTCAAGTA  ACAACCGCGA AAAAGTTGCG CGGAGGAGTT GTGTTTGTGG ACGAAGTACC  GAAAGGTCTT ACCGGAAAAC TCGACGCAAG AAAAATCAGA GAGATCCTCA  TAAAGGCCAA GAAGGGCGGA AAGATCGCCG TGTAATTCTA GAGTCGGGGC  GGCCGGCCGC TTCGAGCAGA CATGATAAGA TACATTGATG AGTTTGGACA  AACCACAACT AGAATGCAGT GAAAAAAATG CTTTATTTGT GAAATTTGTG  ATGCTATTGC TTTATTTGTA ACCATTATAA GCTGCAATAA ACAAGTTAAC  AACAACAATT GCATTCATTT TATGTTTCAG GTTCAGGGGG AGGTGTGGGA  GGTTTTTTAA AGCAAGTAAA ACCTCTACAA ATGTGGTAAA ATCGATAAGG  ATCCGTCGAC CGATGCCCTT GAGAGCCTTC AACCCAGTCA GCTCCTTCCG  GTGGGCGCGG GGCATGACTA TCGTCGCCGC ACTTATGACT GTCTTCTTTA  TCATGCAACT CGTAGGACAG GTGCCGGCAG CGCTCTTCCG CTTCCTCGCT  CACTGACTCG CTGCGCTCGG TCGTTCGGCT GCGGCGAGCG GTATCAGCTC  ACTCAAAGGC GGTAATACGG TTATCCACAG AATCAGGGGA TAACGCAGGA  AAGAACATGT GAGCAAAAGG CCAGCAAAAG GCCAGGAACC GTAAAAAGGC  CGCGTTGCTG GCGTTTTTCC ATAGGCTCCG CCCCCCTGAC GAGCATCACA  AAAATCGACG CTCAAGTCAG AGGTGGCGAA ACCCGACAGG ACTATAAAGA  TACCAGGCGT TTCCCCCTGG AAGCTCCCTC GTGCGCTCTC CTGTTCCGAC  CCTGCCGCTT ACCGGATACC TGTCCGCCTT TCTCCCTTCG GGAAGCGTGG  CGCTTTCTCA TAGCTCACGC TGTAGGTATC TCAGTTCGGT GTAGGTCGTT  CGCTCCAAGC TGGGCTGTGT GCACGAACCC CCCGTTCAGC CCGACCGCTG  CGCCTTATCC GGTAACTATC GTCTTGAGTC CAACCCGGTA AGACACGACT  TATCGCCACT GGCAGCAGCC ACTGGTAACA GGATTAGCAG AGCGAGGTAT  GTAGGCGGTG CTACAGAGTT CTTGAAGTGG TGGCCTAACT ACGGCTACAC  TAGAAGAACA GTATTTGGTA TCTGCGCTCT GCTGAAGCCA GTTACCTTCG  GAAAAAGAGT TGGTAGCTCT TGATCCGGCA AACAAACCAC CGCTGGTAGC  GGTGGTTTTT TTGTTTGCAA GCAGCAGATT ACGCGCAGAA AAAAAGGATC  TCAAGAAGAT CCTTTGATCT TTTCTACGGG GTCTGACGCT CAGTGGAACG  AAAACTCACG TTAAGGGATT TTGGTCATGA GATTATCAAA AAGGATCTTC  ACCTAGATCC TTTTAAATTA AAAATGAAGT TTTAAATCAA TCTAAAGTAT  ATATGAGTAA ACTTGGTCTG ACAGTTACCA ATGCTTAATC AGTGAGGCAC  CTATCTCAGC GATCTGTCTA TTTCGTTCAT CCATAGTTGC CTGACTCCCC  GTCGTGTAGA TAACTACGAT ACGGGAGGGC TTACCATCTG GCCCCAGTGC  TGCAATGATA CCGCGAGACC CACGCTCACC GGCTCCAGAT TTATCAGCAA  TAAACCAGCC AGCCGGAAGG GCCGAGCGCA GAAGTGGTCC TGCAACTTTA  TCCGCCTCCA TCCAGTCTAT TAATTGTTGC CGGGAAGCTA GAGTAAGTAG  TTCGCCAGTT AATAGTTTGC GCAACGTTGT TGCCATTGCT ACAGGCATCG  TGGTGTCACG CTCGTCGTTT GGTATGGCTT CATTCAGCTC CGGTTCCCAA  CGATCAAGGC GAGTTACATG ATCCCCCATG TTGTGCAAAA AAGCGGTTAG  CTCCTTCGGT CCTCCGATCG TTGTCAGAAG TAAGTTGGCC GCAGTGTTAT  CACTCATGGT TATGGCAGCA CTGCATAATT CTCTTACTGT CATGCCATCC  GTAAGATGCT TTTCTGTGAC TGGTGAGTAC TCAACCAAGT CATTCTGAGA  ATAGTGTATG CGGCGACCGA GTTGCTCTTG CCCGGCGTCA ATACGGGATA  ATACCGCGCC ACATAGCAGA ACTTTAAAAG TGCTCATCAT TGGAAAACGT  TCTTCGGGGC GAAAACTCTC AAGGATCTTA CCGCTGTTGA GATCCAGTTC  GATGTAACCC ACTCGTGCAC CCAACTGATC TTCAGCATCT TTTACTTTCA  CCAGCGTTTC TGGGTGAGCA AAAACAGGAA GGCAAAATGC CGCAAAAAAG  GGAATAAGGG CGACACGGAA ATGTTGAATA CTCATACTCT TCCTTTTTCA  ATATTATTGA AGCATTTATC AGGGTTATTG TCTCATGAGC GGATACATAT  TTGAATGTAT TTAGAAAAAT AAACAAATAG GGGTTCCGCG CACATTTCCC  CGAAAAGTGC CACCTGACGC GCCCTGTAGC GGCGCATTAA GCGCGGCGGG  TGTGGTGGTT ACGCGCAGCG TGACCGCTAC ACTTGCCAGC GCCCTAGCGC  CCGCTCCTTT CGCTTTCTTC CCTTCCTTTC TCGCCACGTT CGCCGGCTTT  CCCCGTCAAG CTCTAAATCG GGGGCTCCCT TTAGGGTTCC GATTTAGTGC  TTTACGGCAC CTCGACCCCA AAAAACTTGA TTAGGGTGAT GGTTCACGTA  GTGGGCCATC GCCCTGATAG ACGGTTTTTC GCCCTTTGAC GTTGGAGTCC  ACGTTCTTTA ATAGTGGACT CTTGTTCCAA ACTGGAACAA CACTCAACCC  TATCTCGGTC TATTCTTTTG ATTTATAAGG GATTTTGCCG ATTTCGGCCT  ATTGGTTAAA AAATGAGCTG ATTTAACAAA AATTTAACGC GAATTTTAAC  AAAATATTAA CGCTTACAAT TTGCCATTCG CCATTCAGGC TGCGCAACTG  TTGGGAAGGG CGATCGGTGC GGGCCTCTTC GCTATTACGC CAGCCCAAGC  TACCATGATA AGTAAGTAAT ATTAAGGTAC GGGAGGTACT TGGAGCGGCC  GCAATAAAAT ATCTTTATTT TCATTACATC TGTGTGTTGG TTTTTTGTGT  GAATCGATAG TACTAACATA CGCTCTCCAT CAAAACAAAA CGAAACAAAA  CAAACTAGCA AAATAGGCTG TCCCCAGTGC AAGTGCAGGT GCCAGAACAT  TTCTCTATCG ATA |
| --- | --- |
